# *IRF7* and *RNH1* are modifying factors of HIV-1 reservoirs: a genome-wide association analysis

**DOI:** 10.1186/s12916-021-02156-5

**Published:** 2021-11-16

**Authors:** Zhenhua Zhang, Wim Trypsteen, Marc Blaauw, Xiaojing Chu, Sofie Rutsaert, Linos Vandekerckhove, Wouter van der Heijden, Jéssica Cristina dos Santos, Cheng-Jian Xu, Morris A. Swertz, Andre van der Ven, Yang Li

**Affiliations:** 1 Department of Internal Medicine and Radboud Center for Infectious Diseases, Radboud University Medical Center, 6525HP Nijmegen, the Netherlands; 2 Department of Genetics, University Medical Center Groningen, 9700RB Groningen, the Netherlands; 3 Genomics Coordination Center, University Medical Center Groningen, 9700RB Groningen, the Netherlands; 4 Department of Computational Biology for Individualised Medicine, Centre for Individualised Infection Medicine, CiiM, a joint venture between the Hannover Medical School and the Helmholtz Centre for Infection Research, Hannover, Germany; 5 HIV Cure Research Center, Department of Internal Medicine, and Pediatrics, Ghent University and Ghent University Hospital, Ghent, Belgium; 6 TWINCORE, Centre for Experimental and Clinical Infection Research, a joint venture between the Hannover Medical School and the Helmholtz Centre for Infection Research, Hannover, Germany

**Keywords:** HIV, Cell-associated HIV-1 RNA, Cell-associated HIV-1 DNA, Genetic variants, Quantitative trait locus

## Abstract

**Background:**

Combination antiretroviral treatment (cART) cannot eradicate HIV-1 from the body due to the establishment of persisting viral reservoirs which are not affected by therapy and reinitiate new rounds of HIV-1 replication after treatment interruption. These HIV-1 reservoirs mainly comprise long-lived resting memory CD4+ T cells and are established early after infection. There is a high variation in the size of these viral reservoirs among virally suppressed individuals. Identification of host factors that contribute to or can explain this observed variation could open avenues for new HIV-1 treatment strategies.

**Methods:**

In this study, we conducted a genome-wide quantitative trait locus (QTL) analysis to probe functionally relevant genetic variants linked to levels of cell-associated (CA) HIV-1 DNA, CA HIV-1 RNA, and RNA:DNA ratio in CD4+ T cells isolated from blood from a cohort of 207 (Caucasian) people living with HIV-1 (PLHIV) on long-term suppressive antiretroviral treatment (median = 6.6 years). CA HIV-1 DNA and CA HIV-1 RNA levels were measured with corresponding droplet digital PCR (ddPCR) assays, and genotype information of 522,455 single-nucleotide variants was retrieved via the Infinium Global Screening array platform.

**Results:**

The analysis resulted in one significant association with CA HIV-1 DNA (rs2613996, *P* < 5 × 10^−8^) and two suggestive associations with RNA:DNA ratio (rs7113204 and rs7817589, *P* < 5 × 10^−7^). Then, we prioritized *PTDSS2*, *IRF7*, *RNH1*, and *DEAF1* as potential HIV-1 reservoir modifiers and validated that higher expressions of *IRF7* and *RNH1* were accompanied by rs7113204-G. Moreover, RNA:DNA ratio, indicating relative HIV-1 transcription activity, was lower in PLHIV carrying this variant.

**Conclusions:**

The presented data suggests that the amount of CA HIV-1 DNA and RNA:DNA ratio can be influenced through *PTDSS2*, *RNH1*, and *IRF7* that were anchored by our genome-wide association analysis. Further, these observations reveal potential host genetic factors affecting the size and transcriptional activity of HIV-1 reservoirs and could indicate new targets for HIV-1 therapeutic strategies.

**Supplementary Information:**

The online version contains supplementary material available at 10.1186/s12916-021-02156-5.

## Background

The introduction of combination antiretroviral treatment (cART) has substantially reduced mortality and increased the life quality of people living with human immunodeficiency virus (HIV) [1]. However, cART does not eradicate HIV-1 from the body while the establishment of persisting viral reservoirs that are not affected by cART, can reinitiate new rounds of HIV-1 replication after treatment interruption. These HIV-1 reservoirs mainly comprise long-lived resting memory CD4+ T cells and are established early after infection [2].

Although early treatment initiation is part of current standard care guidelines, there is high variability in the size and transcriptional activity of the viral reservoir among virally suppressed individuals which is associated with several clinical parameters such as pre-ART viral load [3], time to viral suppression [3], CD4 nadir [4], cART initiation timing [5, 6], and the number viral blips under cART [3, 7]. Hence, identifying host (genetic) factors that contribute to or can explain this observed variation could lead to new (personalized) HIV-1 treatment strategies.

Since the mid-1990s, host genes and genetic variants associated with HIV-1-related phenotypes have been extensively explored. Firstly, genetic determinants of HIV-1 control in untreated PLHIV were analyzed and genome-wide association studies (GWAS) identified the importance of the human leukocyte antigen (HLA) locus [8–12]. Next, several genome-wide association studies (GWAS) have investigated host genetic associations with HIV-1 reservoir size proxied by total HIV-1 DNA in peripheral blood mononuclear cells (PBMCs) and identified modifying genetic variants in major histocompatibility complex (MHC) regions [13, 14]. However, limited work was performed on genetic associations with cell-associated HIV-1 RNA (CA HIV-1 RNA) levels and the ratio of CA HIV-1 RNA to CA HIV-1 DNA (RNA:DNA ratio), the latter indicating (relative) HIV-1 reservoir transcription levels [15].

In this study, we hypothesized that host genetic factors can influence (relative) viral transcription activity in long-term treated PLHIV. To test the hypothesis, we conducted a genome-wide quantitative trait locus (QTL) analysis of CA HIV-1 DNA, CA HIV-1 RNA (transcription activity), and RNA:DNA ratio (relative transcription activity) in CD4+ T cells from a cohort of 207 (Caucasians) PLHIV under long-term suppressive cART (median = 6.6 years) to probe functionally relevant genetic variants.

## Methods

### Participants

A cohort of 207 Caucasian HIV-infected individuals on suppressive cART was included in this study as part of a human functional genomics project (www.humanfunctionalgenomics.org) [16]. The subjects were randomly recruited from the HIV clinic at Radboudumc according to in- and exclusion criteria. Inclusion criteria were: Caucasian ethnicity, age ≥ 18 years, receiving cART > 6 months, and latest HIV-RNA levels < 200 copies/ml. Exclusion criteria were as follows: signs of acute or opportunistic infections, antibiotic use < 1 month 48 prior to the study visit, or active hepatitis B/C. Subject characteristics are summarized in Table 1. Based on the following criteria: less than 6-month cART (4), HIV-2 infection (1), no suppressed viral load (2), no CA HIV-1 DNA or RNA measurement available (1), we excluded another 8 individuals and finalized a 207 PLHIV cohort for downstream analyses.

**Table 1:** Patient characteristics

	*N* = 207
Age, median years (IQR)	52.50 [13.20]
Male sex (%)	190 (91.8%)
HIV diagnosis, median years [IQR]	8.31 [9.50]
cART duration, median years [IQR]	6.60 [7.80]
Plasma viral load < 40 copies/ml, *n* (%)	201 (97.1%)
Absolute CD4 T cell count nadir, median cells/μl (IQR)	250 [230]
Absolute CD4 T cell count at admission, median cells/μl (IQR)	650 [340]
Cell-associated HIV-1 RNA, median log_10_ copies/10^6^ CD4 cells (IQR)	2.20 [0.59]
Cell-associated HIV-1 DNA, median log_10_ copies/10^6^ CD4 cells (IQR)	3.19 [0.63]
Cell-associated HIV-1 RNA/ DNA median ratio (IQR)	0.112 [0.12]

Abbreviations: *cART* combined antiretroviral therapy

### Quantification of cell-associated HIV-1 DNA and HIV-1 RNA from CD4+ T cells

Total CA HIV-1 DNA and CA HIV-1 RNA were measured in 219 samples from patients infected with HIV-1. Frozen PBMCs were thawed and CD4+ T cells were isolated using EasySep Human CD4+ T Cell Isolation Kit (Stemcell Technologies, Vancouver, Canada). CD4+ T cells were aliquoted in two and genomic DNA and RNA were extracted in parallel using the DNeasy Blood & Tissue kit (Qiagen, Hilden, Germany), respetively, using the manufacturer’s protocol with an additional step of using 75 μl elution buffer on the column heated at 56 °C for 10 min, and by Innuprep RNA kit eluting in 30 μl buffer (Westburg, Leusden, The Netherlands). Total RNA was reverse transcribed to cDNA by qScript cDNA SuperMix according to the manufacturer’s protocol (Quantabio, Beverly, MA, USA). Subsequently, HIV-1 DNA and CA HIV-1 RNA were measured by ddPCR (Bio-Rad). Before PCR amplification, 8.65 μl of genomic DNA was restricted by EcoRI (Promega) in a total volume of 10 μl restriction digest for a minimum of 1 h at room temperature. Total HIV-1 DNA and HIV-1 RNA were measured by adding respectively 2 μl and 4 μl in triplicates to ddPCR mix containing 10 μl 2× ddPCR Supermix for Probes, 500 nM primers, and 300 nM probe. DNA was amplified by PCR with an initial denaturation step of 10 min at 95 °C, followed by a denaturation step for 30 s at 95 °C and an annealing/elongation step for 1 min at 56 °C for 40 cycles and a final step of 10 min at 98 °C. Total HIV-1 DNA was normalized by measuring the reference gene RPP30 in duplicate by ddPCR and expressed per million PBMCs. Droplets were read by QX200 droplet reader (Bio-Rad) and automatic threshold setting was done using ddpcRquant software [17]. For HIV-1 RNA normalization, three reference genes per patient, *B2M*, *GAPDH*, and *ACTB* were measured with LightCycler 480 SYBR Green I Master mix. Copies HIV-1 RNA was divided by the geometric mean of the reference genes and expressed per million cells by dividing by the theoretical number of cells per μl RNA.

### Genotype and imputations

#### Genotypes and quality control

DNA obtained from the study cohort was genotyped using the commercially available Infinium® Global Screening Array. Quality controls were applied per sample to exclude zero samples with a call rate ≤ 0.90 and sex mismatch, quality control per SNP to exclude variants with a call rate ≤ of 0.90 and a MAF ≤ 0.001. Four ethnic outliers were filtered out by merging multidimensional scaling plots of samples with 1000 Genomes data, and these outliers were excluded from further analysis. There were no related individuals with identity by descent higher than 0.185 excluded. Between any two related samples, the sample with more missing data was removed. The quality control filters resulted in a data set of 215 samples containing genotype information of 522,455 variants for further imputation.

#### Imputation and quality control

The strands and variants-identifiers were aligned to HRC 1.1 using HRC-1000G-check-bim.pl provided by Michigan Imputation Server [18]. The data were phased using Eagle v2.4 using HRC 1.1 2016 hg 2019 reference panel as a reference panel. Without R^2^ filter, using hg19 as array build, Eagle v2.4 as phasing tool, European population, and we performed both quality control and imputation by the Michigan Imputation Server. After imputation, variants with MAF < 0.1 and imputation quality score of *R*^2^ < 0.3 were excluded. The final imputed data set consisted of 215 individuals and 4,307,246 variants for downstream analyses.

### QTL mapping analysis and function annotation

#### Data preparation and preprocessing

Three traits were either measured or calculated based on measured values. Two of them, quantities of CA HIV-1 DNA and CA HIV-1 RNA per 1 × 10^−6^ CD4+ T cells, were measured and log-transformed to smooth the skewness of raw data. As the third trait, a ratio of CA HIV-1 RNA to CA HIV-1 DNA was calculated and log-transformed. In total, phenotypes were generated from 219 individuals. Four covariables were taken into consideration to correct the linear regression models. Concretely, sex was encoded as 0 and 1 for males and females, respectively. Age was represented by years (months and days were divided into decimal numbers). We also obtained each individual’s duration of known HIV-1 infection which also transformed into years and CD4 nadir which represents the lowest counts of CD4 T cells. The final four-covariable set consisting of 211 individuals was acquired. Finally, by intersecting the individuals with genotypes, phenotypes, and covariables, a final data set including 207 individuals was prepared for the QTL mapping analysis.

#### Genome-wide QTL analysis

The standard additive linear regression models were constructed by MatrixEQTL (version 2.3) [19] to investigate the contribution of genome-wide genotypes to the three traits. Since there are only three phenotypes and they are highly correlated with each other, *P* < 5 × 10^−8^ and *P* < 5 × 10^−7^ were used as the genome-wide significance and suggestive thresholds, respectively. The models were corrected by age, sex, CD4 nadir, and duration of known HIV-1 infection. To investigate genome-wide significant associations, summary statistics for each phenotype were uploaded to the LocusZoom server to visualize regional QTL mapping scan results. Genes in 500 K base-pair up- and downstream to the top SNPs were included in the LocusZoom plots [20]. Manhattan plots and Q-Q plots were drawn by package qqman (version 0.1.4) [21]. Analysis using MatrixEQTL and qqman were performed by in-house scripts in the R programming language (version 3.5.1).

### LD-base estimation and clumping

SNPs with *P* < 5 × 10^−8^ were pooled then the pair-wise LD were calculated based on the reference population (EUR from 1000 genome project phase 3). Any SNP with LD *R*^2^ < 0.6 to the lead SNP was taken as an independent suggestive SNP. Based on each independent suggestive SNP, blocks of SNP with LD R^2^ ≥ 0.6 were identified as LD blocks. The 636 SNPs downloaded from GWAS catalog were clumped by PLINK [22] tool (v1.90b6.18 64-bit) using the following parameters: --clump-p1 5e-8 --clump-p2 1 --clump-r2 0.6 --clump-kb 500. The command reduced the SNPs from 636 to 41 independent SNPs.

### Functional annotations for variants using VEP, HaploReg, RegulomeDB, and SpliceAI

The consequence of variants was annotated by Variant Effect Predictor (VEP) [23], release 90.5, using GRCh37 as a reference and default parameter settings: *--af --appris --biotype --buffer_size 500 --check_existing --distance 5000 --mane --polyphen b --pubmed --regulatory --sift b --species homo_sapiens --symbol --transcript_version --tsl --cache*. To explore the non-coding variants, HaploReg [24] and RegulomeDB [25] were used together by exploiting the LD information of haplotype blocks from the 1000 Genomes Project. HaploReg v4.1 (update 2015.11.05) was used to identify genetic loci that are linked to identified QTL SNPs by *R*^2^ ≥ 0.6 in European populations. By assigning protein binding and chromatin state annotations (from Roadmap Epigenomics and ENCODE projects) to linked SNPs and indels, effects of SNPs on regulatory motifs, expression, and sequence conservation were estimated. As a complementary, RegulomeDB was used to explore regulatory elements including DNase hypersensitivity, biding sites of transcription factors, and promoter regions in non-coding genome regions. SpliceAI [26] online version was used to explore the potential role of identified SNPs in alternative splicing using default settings (Genome version “hg19”, Score type “raw”, Max distance “50”).

### Functional mapping and annotation by FUMA

To understand the identified associations and prioritize gene loci that are potentially causal to the traits, FUMA (v1.3.5e) was used to analyze the summary statistics obtained from the QTL mapping analysis [27]. In brief, summary statistics of each QTL mapping analysis were uploaded and first analyzed by the “snp2gene” function. By this function, independent significant SNPs were identified by *p* value threshold 5 × 10^−7^ and *R*^2^ ≥ 0.6 (comparing to index SNPs). Then, candidate genes were prioritized for each independent significant SNPs, respectively. Correspondingly, using the FUMA tool, we attributed our QTLs to gene based on (1) positional mapping to identify genes that are close to the QTL SNPs; (2) expression QTLs (eQTL) mapping to check whether our QTL SNPs affect the expression of candidate genes; (3) chromatin interaction mapping to estimate whether the SNP involves in modulating gene expression epigenetically. Next, mapped genes were filtered and prioritized by default parameters, and further function annotations were conducted by the “gene2func” function on selected protein-coding genes.

### RNA isolation and qPCR

RNA was extracted from the whole blood of patients infected by HIV-1 using the QIAGEN PAXgene Blood RNA extraction kit (QIAGEN, Netherlands) according to the instructions of the manufacturer. Subsequently, RNA was reversely transcribed into cDNA by iScript (Bio-Rad, Hercules, CA, USA). Diluted cDNA was used for qPCR that was done by using the StepOnePlus sequence detection systems (Applied Biosystems, Foster City, CA, USA) with SYBR Green Mastermix (Applied Biosystems). The mRNA relative expression analysis was done with the 2^−dCt^ method and normalized against the housekeeping gene *RPL37A*. Primer sequences are listed in Additional file 1: Table S1.

### PBMC stimulation and cytokine assessment

Venous blood of patients infected with HIV-1 was collected into 10-mL EDTA tubes (Monoject). PBMC isolation was performed by density centrifugation of blood diluted 1:1 in pyrogen-free PBS over Ficoll-Paque (GE healthcare, UK). After isolation, the cells were resuspended in RPMI culture medium (Roswell Park Memorial Institute medium, Invitrogen, CA, USA) supplemented with 50 mg/mL gentamicin (Centrafarm), 2 mM glutamax (GIBCO), and 1 mM pyruvate (GIBCO) supplemented with 10% human pooled serum. 0.5 × 10^6^ PBMCs in a 100 μL volume were added to round-bottom 96-well plates (Greiner Bio-One, Frickenhausen Germany). Cells were incubated either 100 μg/mL of polyinosinic: polycytidylic acid (Poly I:C), a TLR3 agonist and 5 μg/mL Imiquimod, a TLR7 agonist for 24 h and 7 days, respectively at 37 °C and 5% CO_2_. Levels of interferon (IFN)-alpha and -gamma were measured in the supernatants using Verikine Human IFN Alpha ELISA kit (PBL Assay Science, Piscataway, NJ, USA) and human IFN-Gamma ELISA kit (R&D Systems), according to the instructions of the manufacturer.

### Metabolomics measurement

Time-of-flight mass spectrometry was used to perform the untargeted metabolomics by injection electrospray. Metabolites of plasma samples from the participants were identified at General Metabolics’ labs according to previous methodology [28]. No restriction (e.g., eating, drinking or smoking) was applied before collecting blood samples from the participants. In a fixed time-frame (8-11 AM), venous blood was collected in sterile 10-ml EDTA BD Vacutainer® tubes and the collected samples were frozen and stored before metabolite identification. Mass-to-charge ratio (ion m/z) was used to identify metabolites. Specifically, the relative ion intensities in counts of interested metabolites (seven types of phosphatidylserines) from 174 participants were selected for downstream analysis.

### Statistics

The statistical analyses were conducted in the R environment of version 3.5.1 with default settings and function parameters if no more information is mentioned here. Subject characteristics were summarized and IRQs of continuous variables were calculated to indicate the variability of the phenotype. Spearman’s *ρ* were calculated by *cor.test* function pair-wisely to estimate the relationships among all subject characteristics included by the current study, and subsequently, the corresponding *p* values were adjusted by *p.adjust* using “fdr” method. Student’s test was performed to estimate the difference of CA HIV-1 DNA, CA HIV-1 RNA, and RNA:DNA ratio between males and females at a significant level of 0.05. A linear model was constructed to evaluate the variance of CA HIV-1 RNA using CA HIV-1 DNA, age, sex, duration of known HIV-1 infection, and CD4 nadir as predictors, and coefficients were estimated using default parameters by *lm* function and default parameters. The gene ontology term enrichment was estimated by the hypergeometric test which is implemented in FUMA [27], and the corresponding *p* values were adjusted using the FDR method which is also implemented internally in FUMA. The differential expression analysis for each candidate gene and cytokines was performed using a two-sample Wilcoxon test (*p* value < 0.05). DESeq2 [29] were used to estimate the differential expression of prioritized genes in the downloaded gene expression data from GEO GSE127468 [30, 31].

For the analysis of this study, the following tools were used: MatrixEQTL (v2.3), qqman (v0.1.4), DESeq2 (version 1.30.1), PLINK (v1.90b6.18 64-bit), FUMA (v1.3.5e), VEP (release 90.5), HaploReg (v4.1), R (version 3.5.1), Python (version 3.6.3). Publicly available in-house scripts for the QTL mapping, statistical analysis, and visualization are publicly available (https://github.com/zhenhua-zhang/zzhang-etal-hiv-reservoir).

## Results

### Study cohort and CA HIV-1 DNA/RNA measurements

**Fig. 1:**
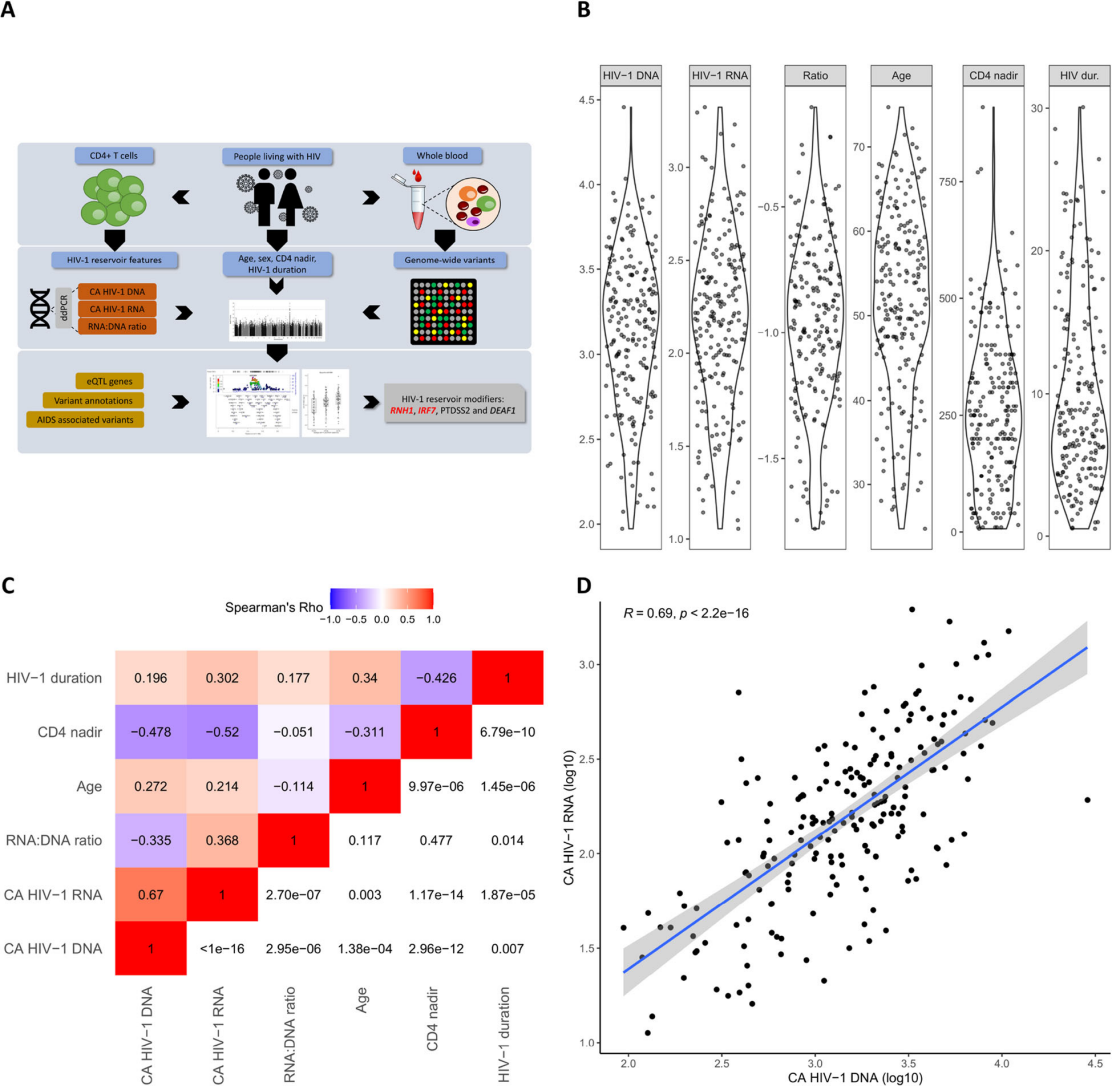
Study design and host characteristics. **A** Overview of the study design. **B** Patient characteristics corresponding to Table 1, including distributions of HIV nucleic acid measurement (CA HIV-1 DNA, CA HIV-1 RNA, and RNA:DNA ratio) and host factors (age, CD4 nadir, and duration of HIV-1 infection). **C** Spearman’s *ρ* among CA HIV-1 DNA, CA HIV-1 RNA, RNA:DNA ratio, and other host factors. The top left triangle shows the *ρ* value, in which the negative and positive correlation values are colored in blue and red, respectively. The bottom right triangle shows the FDR of each correlation analysis. **D** Correlation between CA HIV-1 RNA and CA HIV-1 DNA. Theoretically, CA HIV-1 RNA is highly correlated to CA HIV-1 DNA, while, in our data, we found they are correlated by a Pearson’s *r* = 0.69 (*P* < 2.0 × 10^−16^)

We measured total CA HIV-1 DNA and CA HIV-1 RNA from 219 Dutch PLHIV using chronic cART (Methods) at the Radboudumc (Nijmegen, The Netherlands) [32] and 215 of them were genotyped successfully. The overview of the study is depicted in Fig. 1A. Patient characteristics are summarized in Fig. 1B and Table 1. In brief, the PLHIV cohort included 190 males and 17 females with a median age of 52.5 years interquartile range (IQR) of 13.2 and the median duration of known HIV-1 infection is 8.31 years (IQR = 9.5). The median CA HIV-1 RNA and DNA was 2.20 log copies (IQR = 0.59) and 3.19 log copies (IQR = 0.63) per million CD4+ T cells, respectively, and the median RNA:DNA ratio was 0.112 (IQR = 0.12).

First, we investigated the correlation among the measured HIV-1 reservoir features. Based on Spearman’s *ρ* analysis, CA HIV-1 RNA showed a positive correlation (*ρ* = 0.67, FDR < 1 × 10^−16^) with CA HIV-1 DNA (Fig. 1C, D) which is in line with previous work [15]. Then, we found they both showed a significant negative correlation with CD4 nadir (*ρ* = − 0.50, FDR = 2.25 × 10^−13^ and *ρ* = − 0.53, FDR = 1.05 × 10^−15^ for CA HIV-1 DNA and CA HIV-1 RNA, respectively), which is consistent with previous results [7, 33]. However, CD4 nadir is not correlated with RNA:DNA ratio (*ρ* = − 0.03, FDR = 0.68), which indicates that the CD4 nadir similarly correlates with both CA HIV-1 DNA and CA HIV-1 RNA levels in CD4+ T cells. Of note, CA HIV-1 RNA, CA HIV-1 DNA, and RNA:DNA ratio show consistently positive correlations with duration of known HIV-1 infection (CA HIV-1 DNA, *ρ* = 0.33, FDR = 1.54 × 10^−5^; CA HIV-1 RNA, *ρ* = 0.18, FDR = 0.02; RNA:DNA ratio, *ρ* = 0.19, FDR = 0.012). Furthermore, age is moderately related to any of the three HIV-1 reservoir traits, while sex does not have a significant impact (Additional file 2: Fig. S1).

Lastly, based on a full model analysis using CA HIV-1 RNA as the response variable, all abovementioned host factors and CA HIV-1 DNA explained 54.45% of the total inter-individual variation of CA HIV-1 RNA. Among all those factors, the level of CA HIV-1 DNA contributes the largest with 47.67% of explained variance. This finding leads to the hypothesis that the rest of the unexplained variation could be due to host genetic factors.

### Genome-wide mapping of genetic variants associated with HIV-1 reservoir measurements

To identify genetic determinants of CA HIV-1 DNA, CA HIV-1 RNA, and RNA:DNA ratio in this PLHIV cohort, we conducted a QTL mapping analysis using a linear model. The genotype information of the subjects was measured by the genome-wide Illumina SNPs array followed by imputation. We filtered the imputed variants using thresholds of minor allele frequency (MAF) ≤ 0.1, imputation quality score *R*^2^ < 0.3, resulting in 4,307,246 SNPs in total.

**Fig. 2:**
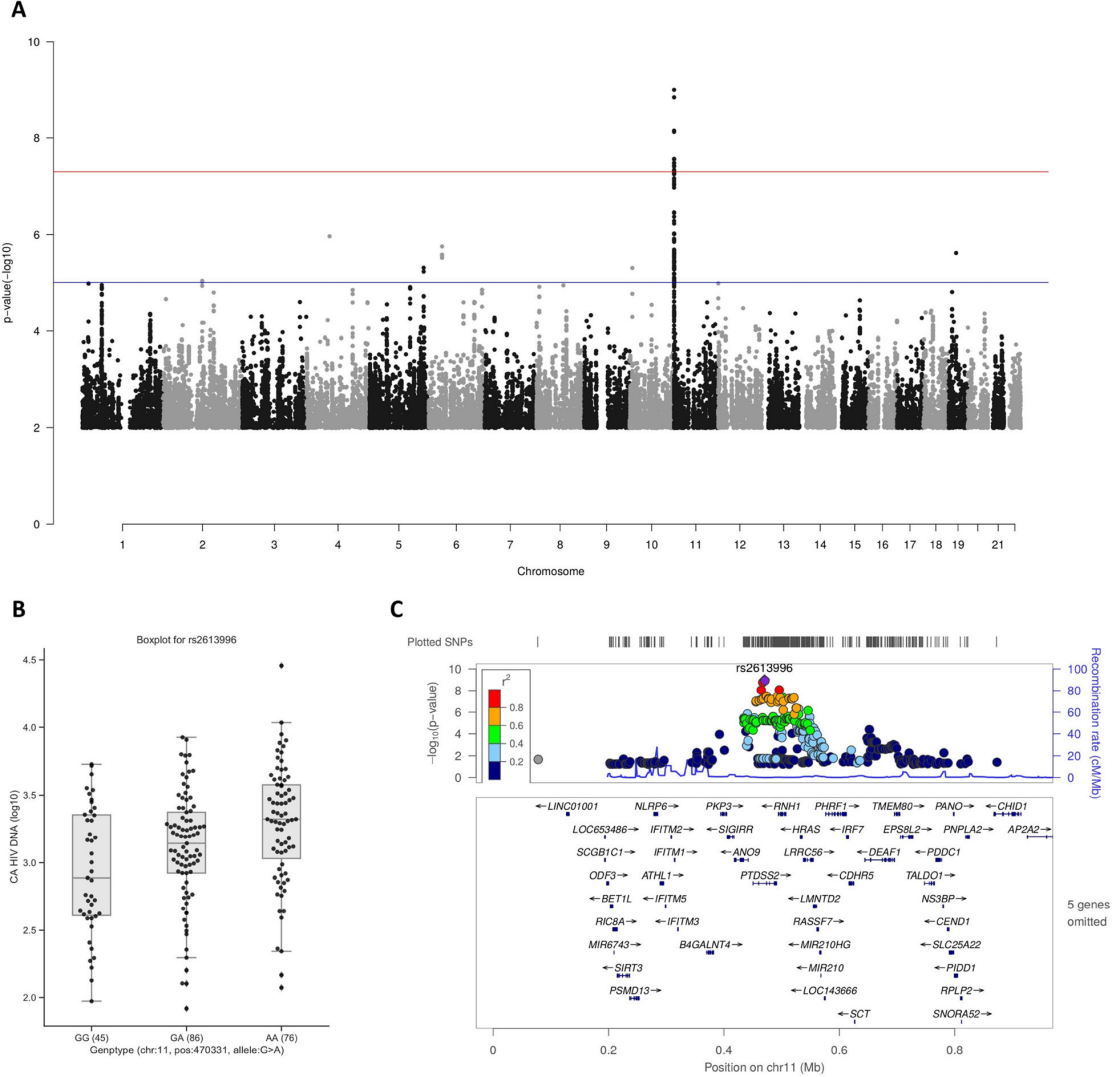
Identified loci associate with CA HIV-1 DNA. **A** Manhattan plot of QTL mapping analysis for CA HIV-1 DNA with CA HIV-1 RNA as one of the covariables. One genome-wide significant locus was identified on the short arm of chromosome 11 with leading SNP rs2613996 at the threshold of *P* < 5 × 10^−8^. **B** Box plot of CA HIV-1 DNA for each genotype of rs2613996. The association *p* value is 9.72 × 10^−10^ and the rs2613996-A allele contributes to CA HIV-1 DNA positively. **C** LocusZooom plot of the top SNP of QTL mapping analysis for CA HIV-1 DNA. Every dot in the figures represents a SNP at a threshold of *P* < 0.01. The top SNP was colored in purple and shaped by a diamond marker. The color of the dots indicates its LD *R*^2^ value to the top SNP

First, for QTL mapping analysis on CA HIV-1 DNA and CA HIV-1 RNA, we applied an additive linear regression model with covariables including age, sex, CD4 nadir, and HIV-1 duration with either CA HIV-1 DNA or CA HIV-1 RNA (Fig. 2A and Additional file 2: Fig. S2A-C). For CA HIV-1 DNA, one genome-wide significant locus with top SNP rs2613996 (Chr11:470331) was identified (*P* < 5 × 10^−8^).

**Fig. 3:**
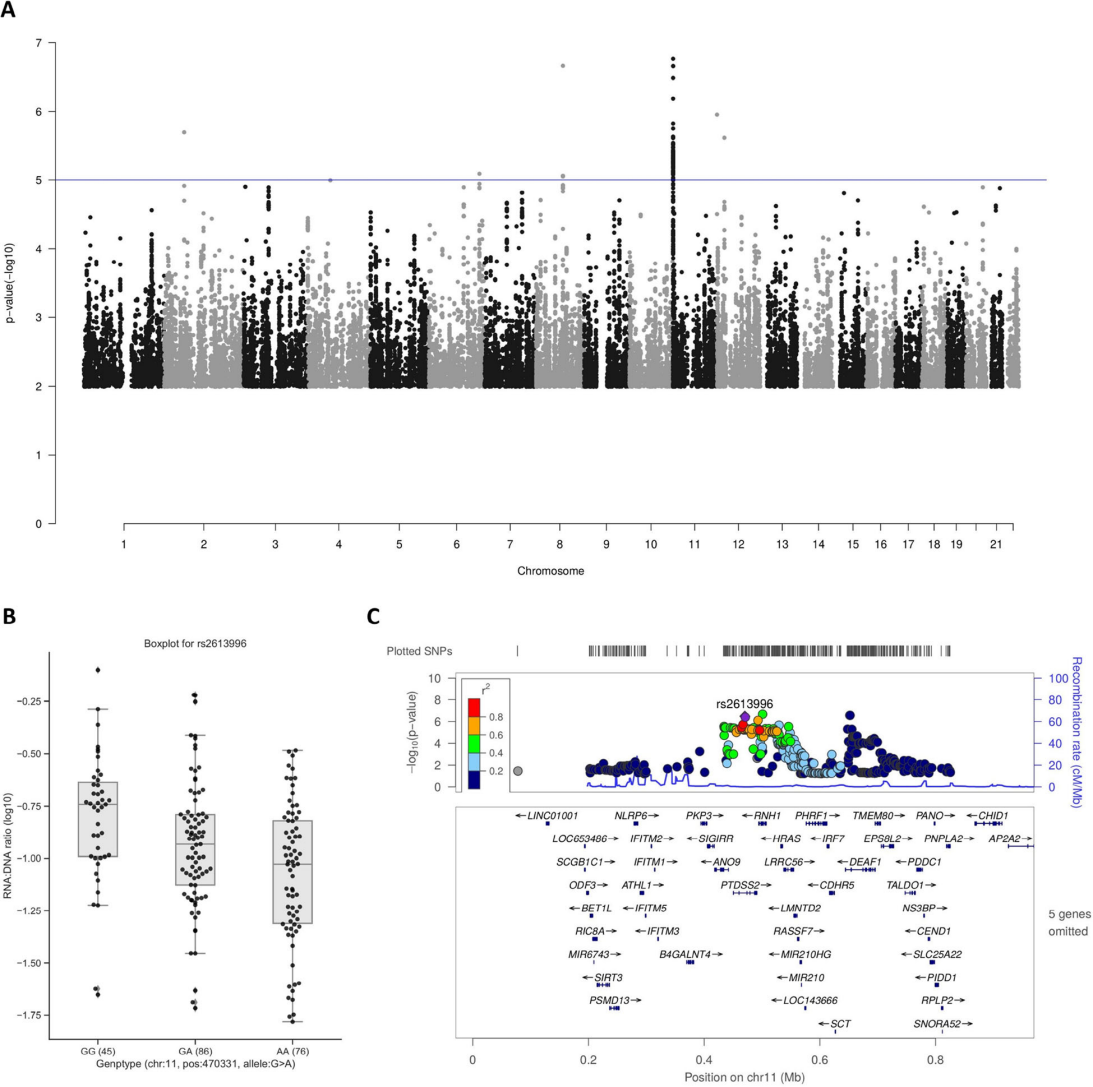
Identified loci associated with RNA:DNA ratio. **A** Manhattan plot of QTL mapping analysis for the RNA:DNA ratio. No genome-wide significant locus was identified at the threshold of *P* < 5 × 10^−8^; however, we identified two suggestive associations with the RNA:DNA ratio at the threshold of *P* < 5 × 10^−7^. **B** Box plot for each genotype of rs2613996 from QTL mapping analysis for the RNA:DNA ratio. The top SNP, rs2613996 is suggestively associated with the trait (*P* = 2.19 × 10^−7^). This SNP is also the top SNP of the locus identified as genome-wide significant association with CA HIV-1 DNA. **C** LocusZoom plot of the independent SNP of QTL mapping analysis for CA HIV-1 DNA on chromosome 11 (rs2613996)

Then, for the HIV-1 RNA:DNA ratio, which has been reported to be a proxy for the relative transcription activity of the reservoir [15, 34], we applied a similar regression model with age, sex, CD4 nadir, and HIV-1 duration as covariables. At a suggestive threshold of *P* < 5 × 10^−7^, we identified two loci (Fig. 3A and Table 2) that are associated with the RNA:DNA ratio at top SNP rs7817589 (Chr8:82154232) and rs7113204 (Chr11:500248), respectively. We also noticed that at the locus by rs7113204, two additional genetic variants, rs2613996 (Chr11:470331) and rs12366210 (Chr11:650994), both show a *P* < 5 × 10^−7^ but they are independent from the top SNP and each other (*R*^2^ < 0.6).

**Table 2:** Identified genetic variants associated with HIV-1 reservoir features

SNP	Chr	Pos.	Allele	Candidate genes	Variant type	Replication	Index SNP	Trait	*P* value	Beta
rs2613996^A^	11	470331	A	*PTDSS2*^B, C^	Regulatory region variant, intronic variant	McLaren et al, 2013	rs2613996	CA HIV-1 DNA	9.72 × 10^-10^	0.18
							rs7113204	RNA:DNA ratio	2.19 × 10^-7^	− 0.284
rs7113204	11	500248	G	*RNH1*^B^, *PTDSS2*^C^, *IRF7*^C^	Intronic variant	–	rs7113204	RNA:DNA ratio	1.72 × 10–^7^	− 0.199
rs12366210	11	650994	G	*DEAF1*^B^	Intronic variant	–	rs7113204	RNA:DNA ratio	3.26 × 10^−7^	− 0.165
rs7817589	8	82154232	C	*RP11-1149 M10.2*, *PAG1*^D^	Upstream gene variant	–	rs7817589	RNA:DNA ratio	2.17 × 10^−7^	0.252

^A^ rs2613996 is the top QTL SNP to CA HIV-1 DNA and also an independent QTL SNP to the RNA:DNA ratio. Also, the SNP is located on the second intron of *PTDSS2*

^B^ The gene where the SNP is located

^C^ Genes that are identified as AIDS-related by hands (e.g., from literature)

^D^ Co-regulated with lncRNA gene *RP11-1149 M10.2*

### Functional annotation of genetic variants associated with CA HIV-1 DNA and RNA:DNA ratio

To explore the functional relevance and biological effects of these three QTLs, we firstly assessed the genetic consequence (e.g., missense mutation) of the identified QTL SNPs using VEP [23]. The CA HIV-1 DNA-associated SNP rs2613996, located in the *PTDSS2* gene, was predicted as a “regulatory region variant,” while the rest of the SNPs were annotated as either “intron variants” or “upstream gene variants” (Table 2). None of the intron variants were identified to be associated with alternative splicing events, according to the SpliceAI online database [26].

We also discovered two synonymous variants by applying HaploReg [24] to explore linked SNPs in haplotype blocks of the identified QTL SNPs. One of the variants, rs10615, which is in strong linkage disequilibrium (LD, *R*^2^ = 0.96, *D*’ = 1) with the RNA:DNA ratio associated QTL SNP rs12366210, is located at the *DEAF1* gene. This gene encodes a transcription factor affecting *HNRPA2B1* that subsequently involves the transport of HIV-1 genomic RNA out of the nucleus [35–37]. The other variant, rs17584, which is in strong LD with rs7113204 (*R*^2^ = 0.81, *D*’ = 1.0), is located within the *RNH1* gene.

Next to these approaches, we applied the FUMA tool [27] to interpret the functional effect of identified QTLs. We identified a list of 66 candidate genes (Additional file 1: Table S2) which showed enrichment for pathways involved in the immune system, lipid metabolism, and regulation of viral entry into host cells (hypergeometric tests, adjusted *P* < 0.05, Additional file 2: Fig. S3). The candidate genes were further prioritized based on literature and potential involvement in HIV-1 processes, resulting in the following four genes for follow-up analysis: *PTDSS2*, *RNH1*, *IRF7*, and *DEAF1* (Table 2).

### CA HIV-1 DNA-associated rs2613996 is correlated with the expression of the *PTDSS2* gene

The genome-wide QTL mapping analysis identified rs2613996 at *PTDSS2* to be associative with CA HIV-1 DNA (*P* = 9.72 × 10^−10^, Table 2, Fig. 2A, C) and RNA:DNA ratio (*P* = 2.19 × 10^−7^, Table 2, Fig. 3A, C). Since the SNP was also implicated as a potential regulatory region variant of *PTDSS2*, we further assessed its association with the expression level of the *PTDSS2*. By probing whole blood eQTL data from healthy donors (eQTLGen Consortium) [38], we found that rs2613996 is an eQTL SNP to *PTDSS2* (*P* = 3.1 × 10^−50^, Additional file 1: Table S3) and importantly the estimated allele (rs2613996-A) was positively correlated with the upregulation of this gene and CA HIV-1 DNA levels simultaneously.

To validate the function of PTDSS2 in HIV-1 reservoir scenario, the intensity of PS was measured using TOF-MS system method for the same cohort. As shown in Additional file 2: Fig. S4A, we estimated the correlation between PS and RNA:DNA ratio using Spearman’s rank-order correlation test, and as expected, RNA:DNA ratio was significantly correlated with two types of PS: HMDB0061555 (*P* = 5.66 × 10^−3^, *ρ* = 0.220) and HMDB0012417 (*P* = 0.014, *ρ* = 0.196). Then, we also tested the difference of PS intensity between individuals grouped by alleles from both rs2613996 and rs7113204. However, for the tested PS, no significant difference across the estimated genotypes was observed (Additional file 2: Fig. S4B and S4C), which is consistent with the RNA expression results of PTDSS2 in current study. These results suggest the potential function of PS in the activity of HIV-1 reservoir, while the intensity of PS was modulated not only by PTDSS2 but other mechanisms.

### Identified variants at *RNH1* and *IRF7* are correlated with loci expression and RNA:DNA ratio

From the QTL mapping, two loci were identified associated with RNA:DNA ratio at a *p* value threshold of 5 × 10^−7^ (Table 2). One of the QTLs was indexed by SNP rs7817589 (*P* = 2.17 × 10^−7^, Table 2, Fig. 3A, Additional file 2: Fig. S5A and S5B) which is also an eQTL SNP associated with the long non-coding RNA (lncRNA) *RP11-1149 M10.2* (*P* = 3.84 × 10^−14^) by eQTLGen Consortium [38]. As limited information is available for this lncRNA, we performed a co-expression network analysis to identify co-regulated genes using GeneNetwork [39]. Here, we ascertained *PAG1* as a gene co-expressing with the identified lncRNA (co-regulation *P* = 5.3 × 10^−18^, Table 2).

**Fig. 4:**
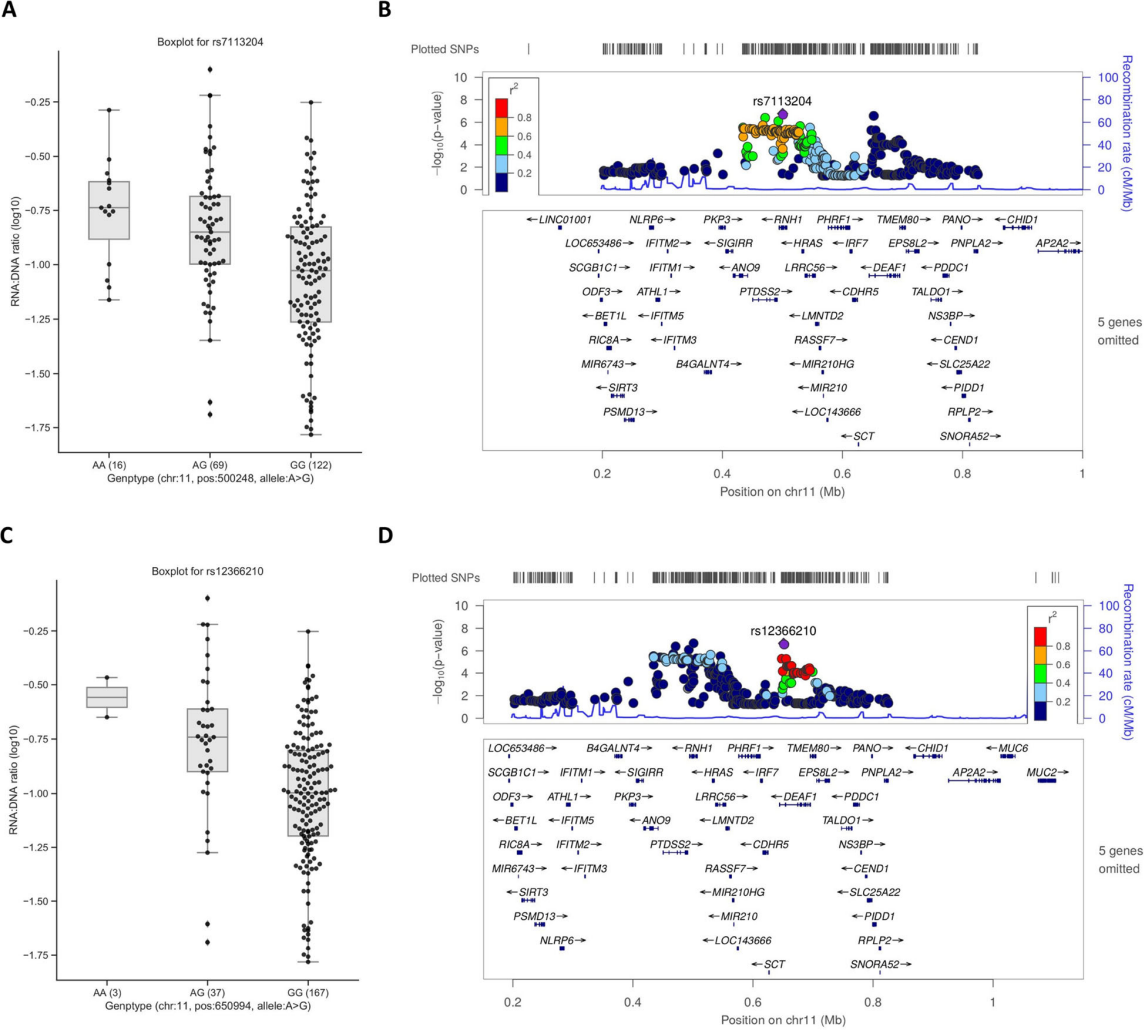
**A** Boxplot for each genotype of rs7113204 from QTL mapping analysis for the RNA:DNA ratio. The estimated association is suggestive at a p value of 1.72 × 10 . The estimated allele is rs7113204-G and is negatively associated with the RNA:DNA ratio. **B** LocusZoom plot of the top SNP of QTL mapping analysis for the RNA:DNA ratio on chromosome 11 (rs7113204). **C** Boxplot for each genotype of rs12366210 from QTL mapping analysis for the RNA:DNA ratio. The rs12366210-G allele of this independent SNP is negatively associated with the RNA:DNA ratio and the SNP is independent of the top SNP of the locus (i.e., rs7113204). **D** LocusZoom plot of the top SNP of QTL mapping analysis for the RNA:DNA ratio on chromosome 11 (rs12366210)

The other locus associated with the RNA:DNA ratio was pinpointed by rs7113204 (*P* = 1.72 × 10^−7^, Table 2, Fig. 4A, B). This SNP is located at the intronic region of *RNH1* which belongs to the family of proteinaceous cytoplasmic RNase inhibitors, and we found that the SNP significantly correlated with the expression of *RNH1* (Additional file 1: Table S3) [38]. We also observed an *IRF7* association with RNA:DNA ratio based on our prioritization methods and knowledge from published work [40]. Interestingly, we found rs7113204-A allele simultaneously suppresses the expression of *IRF7* (Additional file 1: Table S3) and RNA:DNA ratio (Fig. 4A) but accompanies by a higher *RNH1* expression. Additionally, as shown in Fig. 4C, D, there is another QTL SNP (rs12366210, *R*^2^ = 0.162 with rs7113204) that is suggestively associated with RNA:DNA ratio (*P* = 3.26 × 10^−7^) and is located in the intron region of *DEAF1* gene. Importantly, the expression of *DEAF1* is associated with this QTL SNP and especially promoted by rs12366210-A allele according to eQTL analysis [38].

### *IRF7* and *RNH1* expression in PBMCs of PLHIV increase with allele rs7113204-G presence

**Fig. 5:**
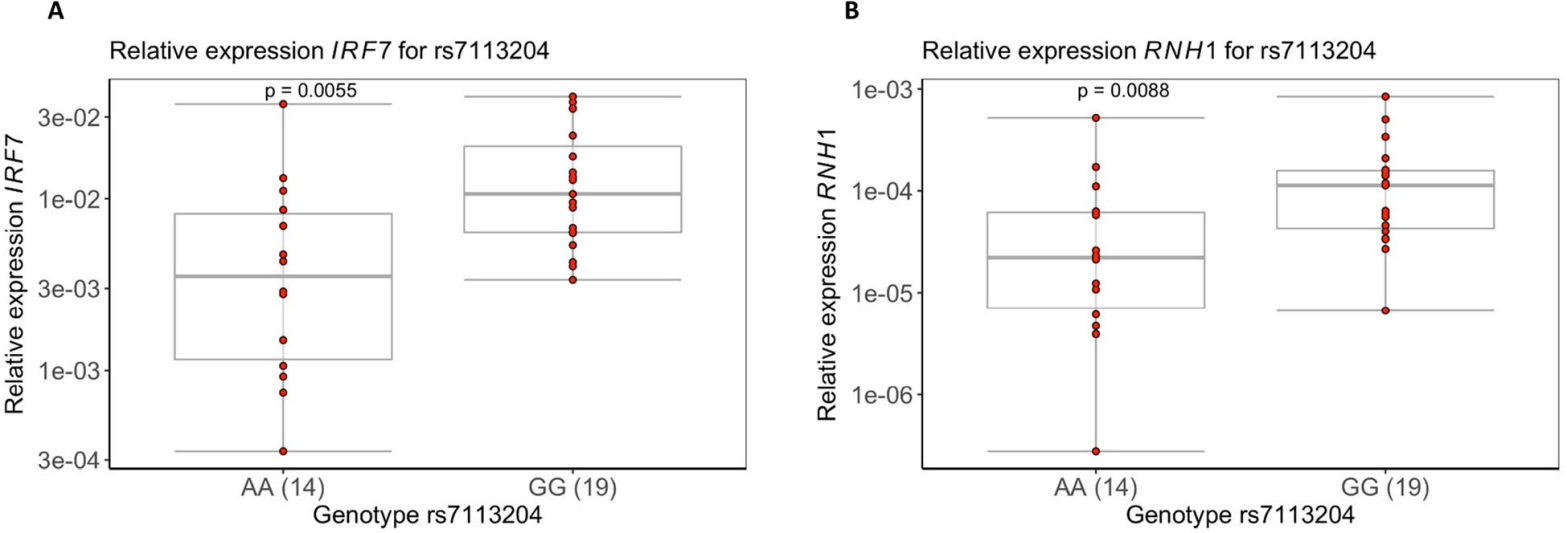
Relative expression of *IRF7* and *RNH1* measured for PLHIV carrying different variants (rs7113204). **A** Box plot of relative expression of *IRF7* compared in a subset of subjects with SNP rs7113204-A (*n* = 14) compared to subjects with SNP rs7113204-G (*n* = 19). **B** Box plot of relative expression of *RNH1* in subjects with SNP rs7113204-A compared to subjects with SNP rs7113204-G. Data in both box plots are visualized in log transformation. Both groups are compared by using the two-sample Wilcoxon test because of the non-normal distribution

To validate candidate genes pinpointed by SNPs associated with CA HIV-1 DNA and RNA:DNA ratio, the RNA expression of *PTDSS2*, *IRF7*, and *RNH1* genes were measured in PBMCs by qPCR. The relative expression of *IRF7* was significantly higher in subjects with rs7113204-G compared with ones carrying rs7113204-A (median [IQR], 0.011 [0.014] vs 0.004 [0.007], *P* < 0.005; Fig. 5A). Moreover, the relative expression of *RNH1* was also significantly higher in subjects with rs7113204-G (1.13 × 10^−5^ [1.139 × 10^−4^] vs 2.22 × 10^−6^ [5.48 × 10^−5^], *P* < 0.05; Fig. 5B). However, we did not observe a significant divergence of relative expression at *PTDSS2* regarding the estimated alleles (Additional file 2: Fig. S6).

### Publicly available gene expression data validated potential functions of prioritized genes

To further estimate the function of prioritized genes in this study, we downloaded RNA-seq read counts for isolated CD4+ T cells which were either infected by the HIV-1 virus or not [30]. Then, we checked if the *DEAF1*, *IRF7*, *PTDSS2*, and *RNH1* were differentially expressed in CD4+ T cells at four time-points: day 3, day 7, day 9, and day 14. As shown in Additional file 2: Fig. S7, *DEAF1* was significantly highly expressed in uninfected samples at day 14 (FDR < 0.05), which indicates an impaired activity of HIV-1 RNA transport and consequently a latent reservoir. This is in line with our findings: rs12366210-A is associated with both higher *DEAF1* expression (eQTL) and higher RNA:DNA ratio. Also, *IRF7* was highly expressed in each time point at a *P* < 0.05 (nominal *p* value), which indicates that it is involved in the whole early infection stages. Similar to *DEAF1*, we observed significant differential expression of *PTDSS2* at day 14, which indicates the potential role of *PTDSS2* after the establishment of the HIV-1 viral reservoir. The *RNH1* displayed a divergence between infected and non-infected CD4+ T cells. In brief, *RNH1* is upregulated at the early stage of infection but downregulated after 2 weeks when comparing its expression in infected CD4+ T cells to non-infected ones. This result indicates *RNH1* functions in the initial infection stage but drops back after the establishment of HIV-1 reservoir.

### Interferon-γ production in PBMCs stimulated with Imiquimod is significantly diverged by the RNA:DNA ratio associated variant

**Fig. 6:**
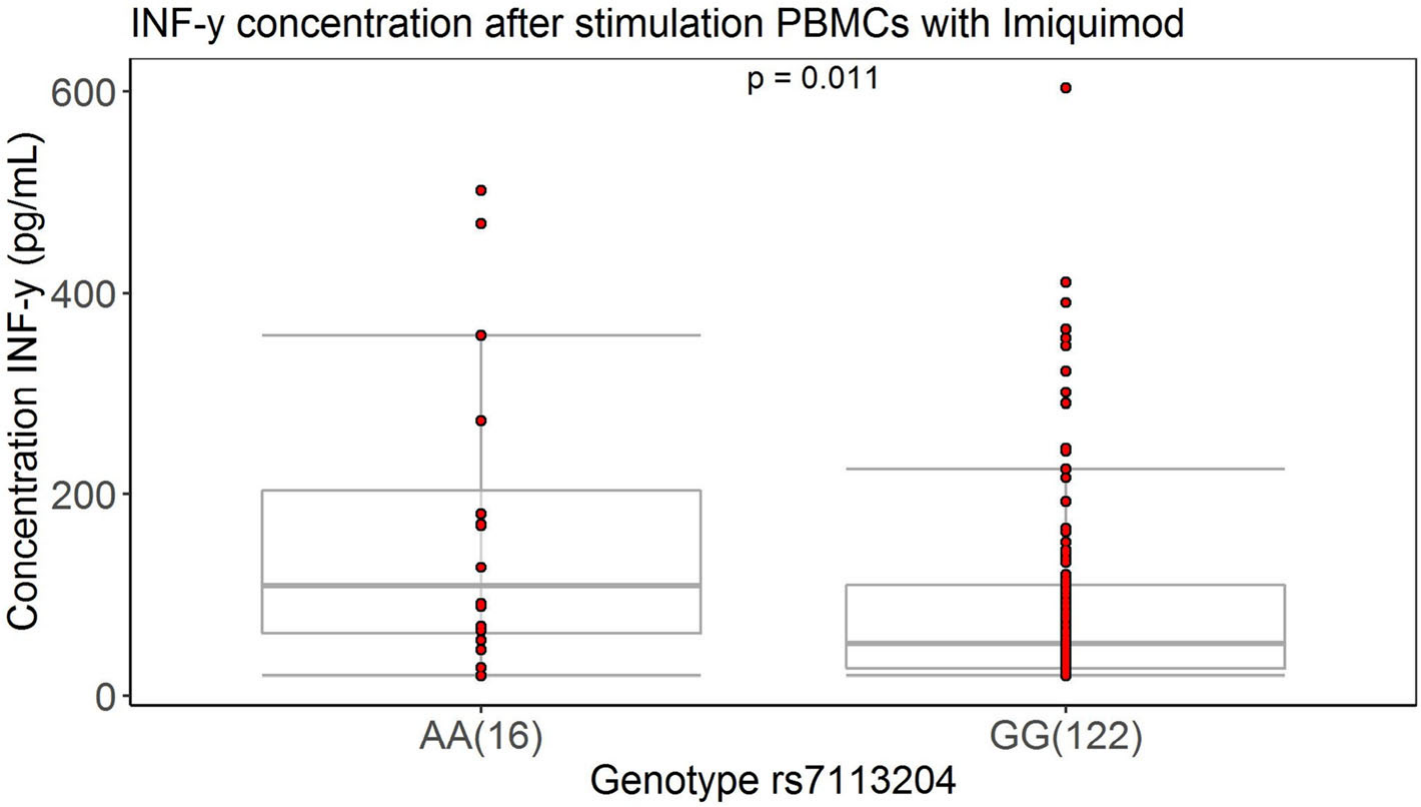
The difference of interferon-gamma production among individuals carrying different alleles of rs7113204. Box plot of interferon-gamma (IFN-γ) production 7 days after imiquimod (TLR7 agonist) stimulation in PBMCs in subjects with SNP rs7113204-A (*n* = 16) and with rs7113204-G (*n* = 122). Groups are compared with two samples Wilcoxon test because of the non-normal distribution

*IRF7* is an essential regulator of type I Interferons (IFN). Pathogen recognition receptors, such as Toll-like receptors (TLR) 3, 7, and 9 [41], may trigger *IRF7* translocalization to the nucleus where, together with other co-activators, it forms a transcriptional complex that activates transcription of targeted genes. Polyinosinic:polycytidylic acid (Poly I:C) is a synthetic analog of double-stranded RNA that is recognized by TLR3. To further validate the role of *IRF7*, PBMCs were stimulated with Poly I:C and the level of interferon-alpha (IFN-α) was subsequently measured in rs7113204-A (*n* = 16) and rs7113204-G (*n* = 121) carriers. No difference was found in IFN-α production between rs7113204-A and rs7113204-G carriers (163.52 pg/mL [159.27] vs 207.00 pg/mL [333.1], *p* = 0.49; Additional file 2: Fig. S8). There is evidence that *IRF7* also plays a role in interferon-gamma (IFN-γ) production [42, 43]. In PBMCs stimulated with Imiquimod (TLR7 agonist), we observed a significant difference in IFN-γ production between rs7113204-A (*n* = 16) and rs7113204-G (*n* = 122) bearers after 7 days (108.56 pg/ml [141.92] vs 51.11 pg/mL [82.59], *p* = 0.011; Fig. 6).

### Shared genetic associations between previous HIV-1 related GWAS and current study

To explore the common host genetic traits among HIV-1 reservoirs and other HIV-1 related phenotypes, summary statistics of publicly available HIV-1 GWAS results (European cohorts) were downloaded from the GWAS catalog [44] and subsequently compared with results from the present study (Additional file 1: Table S4). We clumped 636 SNPs from the downloaded summary statistics by PLINK [22] (Methods) and produced 41 independent SNPs with *P* < 5 × 10^−8^. By probing the acquired independent SNPs in our summary statistics, we found six shared SNPs at a nominal *p* value threshold of 0.05 in our study cohort (Additional file 1: Table S4). Among all the shared SNPs, one SNP, rs9264942 (*P* = 0.022, Chr6:31274380) that presented in our association analysis was reported as one of the genome-wide significant SNPs in a GWAS study of HIV-1 viral load [10]. Notably, the variant pinpoints the *HLA-C* that is broadly reported as an important locus associated with HIV-1 disease progression [45]. Another shared SNP in CA HIV-1 DNA analysis is rs1015164 (*P* = 0.03, Chr3:46451680), and the SNP was reported in an across-ethic cohort of HIV-1 controllers and progressors [46]. Importantly, the *CCR5* that is highlighted by the SNP is well known as an HIV-1 co-receptor that is crucial for the CCR5-tropic virus to infect T cells. Moreover, the rs7568498 SNP in our QTL analysis for RNA:DNA ratio (*P* = 0.024, Chr2:162029113, *TANK*) was also reported as a genome-wide significant SNP in a Hispanic patient cohort by Leger and colleagues [47]. More information could be found for the three SNPs in Additional file 1: Table S4.

To confirm our findings in published QTL studies, we requested the summary statistics of a comprehensive meta-analysis on HIV-1 viral load by McLaren et al. [48]. By probing independent SNPs (*P* < 5 × 10^−7^) from our results in the meta-analysis results, we were able to replicate one QTL SNP (rs2613996, *P* = 6.5 × 10^−3^), which is significantly associated with viral loads after Bonferroni correction (*P* < 0.01), associated with CA HIV-1 DNA and RNA:DNA ratio (Table 2).

## Discussion

In this study, CA HIV-1 DNA and RNA levels were determined in a PLHIV cohort on long-term cART and used as a starting point for evaluating host genetic variants that could be linked to the size and transcriptional activity of the HIV-1 reservoirs. In short, we performed genome-wide QTL mapping analyses for these HIV-1 reservoir features and included functional evaluation of the identified genetic variants to assess their correlation with the expression of identified genes. Overall, we identified one genome-wide significant genetic locus that is associated with CA HIV-1 DNA corrected by CA HIV-1 RNA (*P* < 5 × 10^−8^) and two loci that are suggestively related to RNA:DNA ratio (*P* < 5 × 10^−7^).

Both CA HIV-1 DNA-associated QTL SNP (rs2613996) and RNA:DNA ratio-related SNP (rs7113204) highlighted *PTDSS2*. This gene encodes phosphatidylserine synthase 2, an enzyme that catalyzes the conversion of phosphatidylethanolamine to phosphatidylserine (PS). PS is a phospholipid that normally resides in the inner leaf of the cell membrane. Cells undergoing programmed cell death (or apoptosis) redistribute PS, which functions as an “eat-me” signal, to the outer leaf of the cell membrane. Subsequently, the signal can be recognized by phagocytes, for instance, macrophages, which clear the dead cells by phagocytosis to circumvent inflammation and autoimmune reactions [49, 50]. PS also acts as an important cofactor that facilitates viral entry in the fusion stage of the HIV-1 life cycle [51]. It is also thought that PS exposed to the infected cell inhibits or facilitates multiple steps in HIV replication [49]. Moreover, the exposure of PS on the outer leaf of the cell membrane broadcasts an eat-me signal and plays a crucial role in the HIV-1 pathogenesis. For instance, macrophages selectively recognize and engulf CD4+ T cells that are infected by HIV-1 or dead or dying. Subsequently, the macrophages could eliminate the virus or be infected by HIV-1 after the endocytosis, where the later contributes to the establishment of latent viral reservoir [52]. Meanwhile, the HIV-1-infected macrophages introduce difficulties to suppress HIV-1 efficiently as the CD8+ cytotoxic T cells are less capable of eliminating HIV-1-infected macrophages comparing to eliminating HIV-1-infected CD4+ T cells, which promotes chronic inflammation and introduces barriers to cure patients [53]. Based on the biological function, it is conceivable that *PTDSS2* can play a role in establishing and maintaining the HIV-1 reservoir. However, we could not validate this by studying the RNA expression of *PTDSS2* via qPCR in subjects with different variants. The relative expression was not statistically different in whole blood samples of these subjects with different variants, which could be due to the small sample sizes. However, for SNP rs7113204, there is a trend of higher relative expression of rs7113204-G compared to rs7113204-A, which corresponds with the results of gene expression levels in eQTL studies [38].

The gene identified by QTL SNP rs7113204 for RNA:DNA ratio is *RNH1* which encodes ribonuclease inhibitor 1. The biological role of ribonuclease inhibitor 1 is not known in its entirety but it is known that it binds diverse proteins in the pancreatic RNase superfamily with high avidity [54]. One of these ligands is the eosinophil-derived neurotoxin (EDN), also known as RNase 2. EDN can inhibit HIV-1 replication in HIV-1-infected cells [55, 56]. EDN can maturate and activate human dendritic cells and enhance innate and antigen-specific immune responses [57]. Theoretically, inhibiting EDN by RNH1 could lead to more HIV-1 replication and dampens the activation of human dendritic cells with less immune activation, and could influence the RNA:DNA ratio. We observed that rs7113204-A correlates with a higher RNA:RNA ratio and thus a higher relative transcription activity of CA HIV-1 DNA. It is reported that rs7113204-G has a suppressive effect on the expression of the *RNH1* gene in healthy populations [38] (Additional file 1: Table S3). In contrast, we found in our cohort that *RNH1* expression in whole blood is significantly higher in PLHIV with rs7113204-G compared to ones with rs7113204-A. Of note, our study is the first to report *RNH1* expression levels in PLHIV with or without this specific genetic variant.

Another candidate by SNP rs7113204 is *IRF7* (Interferon regulating factor 7) that is known to play a key role in the production of IFN-α in response to viral infections [41]. We observed that the relative expression of *IRF7* was significantly higher in subjects with the rs7113204-G compared to those carrying rs7113204-A, a finding in agreement with eQTL results in healthy populations [38]. Furthermore, our data indicated that subjects carrying the rs7113204-G variant had less relative HIV-1 transcription activity as indicated by lower RNA:DNA ratios. We hypothesized that this lower HIV-1 replication activity may be due to increased type 1 interferon production capacity but we found no difference in *ex vivo* IFN-α production when stimulation PBMCs of subjects with different variants with the TLR3 ligand poly I:C. However, TLR3 binding can induce both IRF3 and IRF7 [58], and the altered IFN-α production could thus be circumvented via IRF3. We therefore also stimulated PBMCs ex vivo with imiquimod, a TLR7 agonist, and found IFN-α concentration below the limit of detection in the supernatants in both groups. This is in contrast to a study that reported detectable IFN-α levels after PBMCs of healthy subjects were stimulated with the same imiquimod concentration [59]. Diminished IFN-α production in plasmacytoid dendritic cells after stimulation with a TLR7 agonist has been reported previously in PLHIV [60, 61], which might explain the undetectable IFN-α levels in both groups. The importance of IRF7 and type 1 interferon signaling pathways was recently also shown in studies exploring the role of RNA deadenylase complex, CNOT, in HIV infection. CNOT knock-outs suppressed HIV replication, by upregulating and type 1 interferon signaling pathways, a phenotype that was rescued by deletion of *IRF7* [62]. Further evidence that IRF7 restricts the establishment of viral latency and viral reactivation was recently confirmed in a study that compared chronic gammaherpesvirus infections in IRF7-deficient mice and wild-type mice [42]. This study also showed higher IFN-γ levels in IRF7-deficient mice, the data from our study point in the same direction as we found higher IFN-γ levels in the supernatant of PBMC of subjects with rs7113204-A variant after stimulation with imiquimod. Also in a murine cytomegalovirus infection model, IRF7-deficient mice had a higher IFN-γ production during acute infection compared to wild-type mice [43]. Previous studies have indeed shown that IFN-γ may play an important role as an inducer of HIV expression [63]. More research is needed to explore the role of IRF7 in HIV latency.

The gene identified by QTL SNP rs12366210 for RNA:DNA ratio is *DEAF1*. Deformed epidermal autoregulatory factor 1 (encoded by *DEAF1*) is a transcription factor that regulates the expression of hnRNP A2/B1 (heterogeneous nuclear ribonucleoprotein A2/B1). The hnRNP A2/B1 plays a role in HIV genomic RNA trafficking out of the nucleus to the cytoplasm [36]. Accumulation of unspliced HIV-1 RNA in the nucleus will be presumably degraded, which could lead to a lower RNA:DNA ratio. Further research is needed to elucidate the role of *DEAF1* in affecting the viral reservoir. We also included the *PAG1* gene as a candidate that is related to the RNA:DNA ratio as it was reported to play an important role in the regulation of T cell activation [64]. Other candidate genes are listed in Supplementary Table 2.

Regarding host genetic effects on HIV-1 reservoir traits, to our knowledge, two similar studies have been conducted to explore the relationship between host genetic traits and the size of HIV-1 reservoirs in the past decades [13, 14]. However, both of them measured CA HIV-1 DNA in PBMCs only, while we determined both CA HIV-1 DNA and RNA from CD4+ T cells, which intensively avoided the confounding of cell proportions. Additionally, the relative transcription activity that is represented by the RNA:DNA ratio gives additional weight to our study by stretching study aspects from static (size of HIV-1 reservoir) to dynamic (relative transcription activity). Of note, in the analysis that linked non-genetic host factors to CA HIV-1 RNA with CA HIV-1 DNA as covariable, the model can explain 54.45% variation of CA HIV-1 RNA exclusively, which indicates that other factors also affect the variations. The unexplained variance could be attributed to (1) the amount of integrated defective proviral DNA, (2) the various transcription rates of the loci in which the HIV-1 genome is integrated, and (3) various decay rates between different patients.

The strength of our study includes the follwoing: (1) we isolated CD4+ T cells, which circumvent the bias introduced by cell proportion when using PBMCs; (2) we estimated the effects of genetic variants not only on CA HIV-1 DNA and CA HIV-1 RNA, but also their corresponding ratio that indicate the relative transcriptional activity; (3) the genetic background was homogeneous as all recruited individuals were Caucasian adults. However, one of the major limitations is the sample size which restrains the power to identify weak or moderate genetic associations with the HIV-1 reservoir features evaluated in this study. Moreover, in the regression analysis, limited covariates were included in the linear model, which could overlook important host and environmental factors that affect HIV-1 reservoir traits. Finally, only Caucasian individuals were included in the analysis and consequently the identified association could be specific in the studied race. Therefore, larger trans-ethic cohorts are desired to capture more associations.

The methods used in the current study are generalizable. For example, CD4+ T cells were isolated by a commercially available isolation kit. Then, the CA HIV-1 DNA and RNA were measured using the well-developed and widely used ddPCR method. For the genome-wide genotyping, the commercially available Infinium® Global Screening Array were used. As to the genome-wide association analysis, the QLT mapping methods and association analysis can be equally applied to any other quantitative trait or disease phenotype, respectively. Finally, the integration with eQTL data and public available RNA-seq data for the prioritization of candidate genes can be applied in future genetic studies. There are also several limitations in this study. The findings from the current study need to be further replicated in a larger independent cohort. As our cohort consists of western European descent, future studies in a different genetic background will be desired.

Altogether our data indicate that *PTDSS2*, *RNH1*, *IRF7*, and *DEAF1* are potential HIV-1 reservoir modifiers. Of note, we showed higher expressions of *IRF7* and *RNH1* in PLHIV carrying the rs7113204-G variant while these subjects also had evidence of lower relative HIV-1 transcription activity.

## Conclusions

This genome-wide QTL analysis identified one significant genetic locus associated with CA HIV-1 DNA (*p* value < 5 × 10^−8^), and four suggestive associations were found for the RNA:DNA ratio (*p* value < 5 × 10^−7^). These associations highlighted four genes that potentially affect the CA HIV-1 DNA and RNA:DNA ratio: *PTDSS2*, *RNH1*, *IRF7*, and *DEAF1*. We found that the expressions of *IRF7* and *RNH1* were significantly correlated with one of the identified variants (rs7113204) using qPCR. Our results suggest *IRF7* and *RNH1* are potential modifying factors of HIV-1 reservoirs and these observations could indicate targets for future therapeutic strategies to lower HIV-1 reservoir size or activity in PLHIV and HIV-1 patients.

## Supplementary Information

**Additional file 1: Table S1**. Primers used in this study for qPCR. **Table S2**. Candidate genes identified by FUMA tool. **Table S3**. QTL SNPs affect the expression of identified loci. **Table S4**. Publicly available AIDS GWAS QTLs estimated in current study

**Additional file 2: Figure S1**. Three panels of the figure are respective differences of CA HIV-1 RNA, CA HIV-1 DNA, and RNA:DNA ratio between male and female. However, by a student test, sex does not affect any of the three traits at the threshold of *P* < 0.05. **Figure S2**. A, B, and C are Manhattan plots for CA HIV-1 DNA, CA HIV-1 RNA, and CA HIV-1 RNA (adjusted by CA HIV-1 DNA). However, no association was found at the threshold of P < 5 × 10^-7^. Moreover, all the associative analyses have an inflation factor close to 1. **Figure S3**. (A) Gene Ontology (biological process) annotation and enrichment of genes at locus identified as genome-wide significantly associated with CA HIV-1 DNA. (B) Gene Ontology (biological process) annotation and enrichment of genes at locus identified as genome-wide suggestively associated with RNA:DNA ratio. **Figure S4**. (A) Correlation between two types of PS and RNA:DNA ratio. The horizontal axis represents the Log_2_ transformed RNA:DA ratio. The vertical axis represents the PS intensity transformed by Log_10_. (B) PS intensity transformed by Log_10_ per genotype of rs7113204. No significant difference among three genotypes was found for each PS. (C) PS intensity transformed by Log_10_ per genotype of rs2613996. No significant difference among three genotypes was found for each PS. **Figure S5**. (A) Box plot for each genotype of rs7817589 from QTL mapping analysis for RNA:DNA ratio. This locus is identified as suggestively associated with the trait at the threshold of *P* = 2.17 × 10^-7^. (B) LocusZoom plot of the top SNP of QTL mapping analysis for RNA:DNA ratio on chromosome 8. **Figure S6**. (A) Box plot of relative expression of *PTDSS2* compared in a subset of subjects with variant rs2613996-A (*n* = 10) compared to subjects with variant rs2613996-G (*n* = 19). (B) Box plot of relative expression of *PTDSS2* in subjects with SNP rs7113204-A (*n* = 11) compared to SNP rs7113204-G (*n* = 14). Data in these box plots are visualized in log transformation. Groups are compared by using the two-sample Wilcoxon test because of the non-normal distribution. **Figure S7**. Relative expression of four prioritized genes (DEAF1, IRF7, PTDSS2, and RNH1). We downloaded gene expression data of *in vitro* primary human CD4+ T cells from GEO GSE127468 and estimated the differential expression of four genes identified by us. The horizontal axis are time-points at which the mRNA were measured and the vertical axis are relative expression quantified by RNA-seq. The red boxes (dots) and blue boxes (dots) represent relative expression level of each gene in HIV-1 infected CD4+ T cells and control CD4+ T cells *in vitro*, respectively. The double stars (**) mark *p*-value < 0.01 and the single star (*) mark *p*-value < 0.05. **Figure S8**. Box plot of interferon-alpha (IFN-α) production 24 hours after poly I:C stimulation in PBMCs in subjects with SNP rs7113204-A (*n* = 16) and with rs7113204-G (*n* = 121). Groups are compared with two samples Wilcoxon test because of the non-normal distribution.

## Data Availability

The datasets supporting the conclusions of this article are included within the article and its additional files. The materials used in the current study and association analysis summary statistics are available on requests. For the analysis of this study, the following tools were used: MatrixEQTL (v2.3), qqman (v0.1.4), PLINK (v1.90b6.18 64-bit), FUMA (v1.3.5e), VEP (release 90.5), HaploReg (v4.1), R (version 3.5.1), Python (version 3.6.3). Publicly available in-house scripts for the QTL mapping, statistical analysis, and visualization are hosted at https://github.com/zhenhua-zhang/zzhang-etal-hiv-reservoir.

## Abbreviations

CA: Cell-associated
cART: Combination of antiretroviral therapy
ddPCR: Droplet digital PCR
ED: Eosinophil-derived neurotoxin
eQTL: Expression quantitative trait locus
FDR: False discovery rate
GWAS: Genome-wide association study
IFN: Interferon
IQR: Interquartile range
LD: Linkage disequilibrium
lncRNA: Long non-coding RNA
MAF: Minor allele frequency
PBMC: Peripheral blood mononuclear cell
PLHIV: People living with HIV
Poly I:C: Polyinosinic:polycytidylic acid
PS: Phosphatidylserine
QTL: Quantitative trait locus
TLR: Toll-like receptors
VEP: Variant effect predictor
