# Supplementary material for: IRF7 and RNH1 are modifying factors of HIV-1 reservoirs: a genome-wide association analysis
Source: BMC Med. 2021 Nov 16;19:282. doi: 10.1186/s12916-021-02156-5 (PMC8594146; doi:10.1186/s12916-021-02156-5)
Supplement: Supplementary file 1 — Additional file 1: Table S1. Primers used in this study for qPCR. Table S2. Candidate genes identified by FUMA tool. Table S3. QTL SNPs affect the expression of identified loci. Table S4. Publicly available AIDS GWAS QTLs estimated in current study [file 12916_2021_2156_MOESM1_ESM.docx]

## Table S1. Primers used in this study for qPCR

| **Gene symbols** | **Forward primer** | **Reverse Primer** |
| --- | --- | --- |
| *RPL37A* | ATTGAAATCAGCCAGCACG | AGGAACCACAGTGCCAGAT |
| *IRF7* | AACTGTGACACCCCCATCTTC | GGTAGATGGTATAGCGTGGGG |
| *RNH1* | CTTCGAGTCAACCCTGCACT | AAGAGGTTGTCGCTGAGGTG |
| *PTDSS2* | TGCCTCATCTACGACCCAGA | ATGCACATCCACCAGTCTCG |

## Table S2. Candidate genes identified by FUMA tool

| **ENSG ID** | **Gene Symbol** | **HIV-1 Measurement** | **Chr** | **Start** | **End** | **Strand** | **pLI** | **ncRVIS** | **ByPM** | **ByEG** | **MinPvalEG** | **ByCI** | **IndSigSNPs** |
| --- | --- | --- | --- | --- | --- | --- | --- | --- | --- | --- | --- | --- | --- |
| ENSG00000171865 | *RNASEH1* | RNA:DNA ratio, CA HIV-1 DNA | 2 | 3592383 | 3606206 | -1 | 0.016416 | 0.170619 | No | Yes | 3.97E-14 | No | rs7113204; rs2613996; rs7113255 |
| ENSG00000104427 | *ZC2HC1A* | RNA:DNA ratio | 8 | 79578282 | 79632000 | 1 | 0.811433 | -1.15334 | No | No | - | Yes | rs7817589 |
| ENSG00000104435 | *STMN2* | RNA:DNA ratio | 8 | 80523049 | 80578410 | 1 | 0.932288 | -0.0809 | No | No | - | Yes | rs7817589 |
| ENSG00000164683 | *HEY1* | RNA:DNA ratio | 8 | 80676245 | 80680098 | -1 | 0.916105 | -0.31935 | No | No | - | Yes | rs7817589 |
| ENSG00000147586 | *MRPS28* | RNA:DNA ratio | 8 | 80830952 | 80942524 | -1 | 0.000355 | -0.44162 | No | No | - | Yes | rs7817589 |
| ENSG00000076554 | *TPD52* | RNA:DNA ratio | 8 | 80870571 | 81143467 | -1 | 0.005112 | -0.60736 | No | No | - | Yes | rs7817589 |
| ENSG00000205189 | *ZBTB10* | RNA:DNA ratio | 8 | 81397854 | 81438500 | 1 | 0.881586 | -2.60461 | No | No | - | Yes | rs7817589 |
| ENSG00000164684 | *ZNF704* | RNA:DNA ratio | 8 | 81540686 | 81787016 | -1 | 0.972642 | -5.09916 | Yes | No | - | Yes | rs7817589 |
| ENSG00000076641 | *PAG1* | RNA:DNA ratio | 8 | 81880045 | 82024303 | -1 | 0.010837 | 0.712765 | Yes | No | - | Yes | rs7817589 |
| ENSG00000164687 | *FABP5* | RNA:DNA ratio | 8 | 82192598 | 82197012 | 1 | 0.095838 | - | Yes | No | - | Yes | rs7817589 |
| ENSG00000147588 | *PMP2* | RNA:DNA ratio | 8 | 82352561 | 82359758 | -1 | 0.033982 | -0.14662 | Yes | No | - | Yes | rs7817589 |
| ENSG00000205186 | *FABP9* | RNA:DNA ratio | 8 | 82370576 | 82373814 | -1 | 0.00034 | - | Yes | No | - | No | rs7817589 |
| ENSG00000170323 | *FABP4* | RNA:DNA ratio | 8 | 82390654 | 82395498 | -1 | 0.004926 | 0.646129 | Yes | No | - | No | rs7817589 |
| ENSG00000197416 | *FABP12* | RNA:DNA ratio | 8 | 82437216 | 82443613 | -1 | 0.002946 | 0.055192 | Yes | No | - | Yes | rs7817589 |
| ENSG00000133731 | *IMPA1* | RNA:DNA ratio | 8 | 82570196 | 82598928 | -1 | 0.017536 | -0.38314 | Yes | No | - | Yes | rs7817589 |
| ENSG00000253598 | *SLC10A5* | RNA:DNA ratio | 8 | 82605842 | 82608409 | -1 | 0.000395 | -0.30119 | Yes | No | - | Yes | rs7817589 |
| ENSG00000104231 | *ZFAND1* | RNA:DNA ratio | 8 | 82613569 | 82645138 | -1 | 0.000588 | 0.360512 | Yes | No | - | No | rs7817589 |
| ENSG00000164695 | *CHMP4C* | RNA:DNA ratio | 8 | 82644669 | 82671750 | 1 | 0.00183 | 0.367933 | Yes | No | - | No | rs7817589 |
| ENSG00000104497 | *SNX16* | RNA:DNA ratio | 8 | 82711816 | 82755101 | -1 | 4.22E-08 | 1.03089 | No | Yes | 3.26E-06 | No | rs7817589 |
| ENSG00000177951 | *BET1L* | RNA:DNA ratio, CA HIV-1 DNA | 11 | 167784 | 207428 | -1 | 0.378368 | 1.585951 | Yes | No | - | Yes | rs7113204; rs2613996; rs12366210; rs7113255 |
| ENSG00000188076 | *SCGB1C1* | RNA:DNA ratio, CA HIV-1 DNA | 11 | 193080 | 194573 | 1 | 3.34E-05 | 0.362499 | Yes | No | - | Yes | rs7113204; rs2613996; rs12366210; rs7113255 |
| ENSG00000177947 | *ODF3* | RNA:DNA ratio, CA HIV-1 DNA | 11 | 196738 | 200261 | 1 | 0.586975 | 1.893755 | Yes | No | - | Yes | rs7113204; rs2613996; rs12366210; rs7113255 |
| ENSG00000177963 | *RIC8A* | RNA:DNA ratio, CA HIV-1 DNA | 11 | 207511 | 215113 | 1 | 0.000379 | -0.34121 | Yes | No | - | Yes | rs7113204; rs2613996; rs12366210; rs7113255 |
| ENSG00000142082 | *SIRT3* | RNA:DNA ratio, CA HIV-1 DNA | 11 | 215458 | 236931 | -1 | 0.000027 | -0.34099 | Yes | No | - | Yes | rs7113204; rs2613996; rs12366210; rs7113255 |
| ENSG00000185627 | *PSMD13* | RNA:DNA ratio, CA HIV-1 DNA | 11 | 236546 | 252983 | 1 | 0.993513 | 1.387777 | Yes | No | - | Yes | rs7113204; rs2613996; rs12366210; rs7113255 |
| ENSG00000174885 | *NLRP6* | RNA:DNA ratio, CA HIV-1 DNA | 11 | 278365 | 285359 | 1 | - | -0.19277 | Yes | No | - | Yes | rs7113204; rs2613996; rs12366210; rs7113255 |
| ENSG00000142102 | *ATHL1* | RNA:DNA ratio, CA HIV-1 DNA | 11 | 289135 | 296107 | 1 | 3.53E-09 | 0.935514 | Yes | Yes | 6.28E-08 | Yes | rs7113204; rs2613996; rs12366210; rs7113255 |
| ENSG00000206013 | *IFITM5* | RNA:DNA ratio, CA HIV-1 DNA | 11 | 298200 | 299526 | -1 | 0.000116 | - | Yes | No | - | No | rs7113204; rs2613996; rs12366210; rs7113255 |
| ENSG00000185201 | *IFITM2* | RNA:DNA ratio, CA HIV-1 DNA | 11 | 307631 | 315272 | 1 | 0.299376 | 2.273666 | Yes | No | - | Yes | rs7113204; rs2613996; rs12366210; rs7113255 |
| ENSG00000185885 | *IFITM1* | RNA:DNA ratio, CA HIV-1 DNA | 11 | 313506 | 315272 | 1 | 0.221686 | -0.30084 | Yes | Yes | 9.01E-09 | No | rs7113204; rs2613996; rs12366210; rs7113255 |
| ENSG00000142089 | *IFITM3* | RNA:DNA ratio, CA HIV-1 DNA | 11 | 319669 | 327537 | -1 | 0.199202 | 2.726229 | Yes | Yes | 1.25E-05 | No | rs7113204; rs2613996; rs12366210; rs7113255 |
| ENSG00000182272 | *B4GALNT4* | RNA:DNA ratio, CA HIV-1 DNA | 11 | 369796 | 382116 | 1 | 0.81693 | - | Yes | Yes | 7.83E-16 | Yes | rs7113204; rs2613996; rs12366210; rs7113255 |
| ENSG00000184363 | *PKP3* | RNA:DNA ratio, CA HIV-1 DNA | 11 | 392614 | 404908 | 1 | 0.19435 | -0.23505 | Yes | Yes | 2.89E-13 | Yes | rs7113204; rs2613996; rs12366210; rs7113255 |
| ENSG00000185187 | *SIGIRR* | RNA:DNA ratio, CA HIV-1 DNA | 11 | 405716 | 417455 | -1 | 0.005309 | -0.43458 | Yes | Yes | 4.18E-09 | Yes | rs7113204; rs2613996; rs12366210; rs7113255 |
| ENSG00000185101 | *ANO9* | RNA:DNA ratio, CA HIV-1 DNA | 11 | 417933 | 442011 | -1 | 4.59E-11 | -0.03725 | Yes | Yes | 8.7E-107 | Yes | rs7113204; rs2613996; rs12366210; rs7113255 |
| ENSG00000174915 | *PTDSS2* | RNA:DNA ratio, CA HIV-1 DNA | 11 | 448268 | 491393 | 1 | 0.000109 | -0.19531 | Yes | Yes | 2.24E-66 | Yes | rs7113204; rs2613996; rs12366210; rs7113255 |
| ENSG00000023191 | *RNH1* | RNA:DNA ratio, CA HIV-1 DNA | 11 | 494512 | 507300 | -1 | 0.000213 | -0.00953 | Yes | Yes | 1.27E-61 | Yes | rs7113204; rs2613996; rs12366210; rs7113255 |
| ENSG00000174775 | *HRAS* | RNA:DNA ratio, CA HIV-1 DNA | 11 | 532242 | 537287 | -1 | 0.007941 | - | Yes | Yes | 3.04E-94 | Yes | rs7113204; rs2613996; rs12366210; rs7113255 |
| ENSG00000161328 | *LRRC56* | RNA:DNA ratio, CA HIV-1 DNA | 11 | 537527 | 554916 | 1 | 0.000111 | 2.12856 | Yes | Yes | 7.6E-304 | Yes | rs7113204; rs2613996; rs12366210; rs7113255 |
| ENSG00000185522 | *C11orf35* | RNA:DNA ratio, CA HIV-1 DNA | 11 | 554855 | 560779 | -1 | 1.19E-17 | - | Yes | Yes | 5.08E-59 | Yes | rs7113204; rs2613996; rs12366210; rs7113255 |
| ENSG00000099849 | *RASSF7* | RNA:DNA ratio, CA HIV-1 DNA | 11 | 560404 | 564021 | 1 | 3.45E-06 | - | Yes | Yes | 3.35E-09 | Yes | rs7113204; rs2613996; rs12366210; rs7113255 |
| ENSG00000070047 | *PHRF1* | RNA:DNA ratio, CA HIV-1 DNA | 11 | 576486 | 612222 | 1 | 0.948919 | - | Yes | Yes | 8.99E-11 | Yes | rs7113204; rs2613996; rs12366210; rs7113255 |
| ENSG00000185507 | *IRF7* | RNA:DNA ratio, CA HIV-1 DNA | 11 | 612553 | 615999 | -1 | 3.31E-07 | -0.85786 | Yes | Yes | 3.43E-16 | Yes | rs7113204; rs2613996; rs12366210; rs7113255 |
| ENSG00000099834 | *CDHR5* | RNA:DNA ratio, CA HIV-1 DNA | 11 | 616565 | 626078 | -1 | 1.31E-10 | 0.549306 | Yes | Yes | 8.97E-06 | No | rs7113204; rs2613996; rs12366210; rs7113255 |
| ENSG00000070031 | *SCT* | RNA:DNA ratio, CA HIV-1 DNA | 11 | 626431 | 627143 | -1 | 0.406651 | - | Yes | No | - | No | rs7113204; rs2613996; rs12366210; rs7113255 |
| ENSG00000069696 | *DRD4* | RNA:DNA ratio, CA HIV-1 DNA | 11 | 637293 | 640706 | 1 | 7.58E-06 | - | Yes | Yes | 1.07E-18 | Yes | rs7113204; rs2613996; rs12366210; rs7113255 |
| ENSG00000177030 | *DEAF1* | RNA:DNA ratio, CA HIV-1 DNA | 11 | 644233 | 706715 | -1 | 0.000193 | - | Yes | Yes | 2.71E-17 | Yes | rs7113204; rs2613996; rs12366210; rs7113255 |
| ENSG00000177106 | *EPS8L2* | RNA:DNA ratio, CA HIV-1 DNA | 11 | 694438 | 727727 | 1 | 0.001827 | - | Yes | Yes | 4.1E-10 | Yes | rs7113204; rs2613996; rs12366210; rs7113255 |
| ENSG00000177042 | *TMEM80* | RNA:DNA ratio, CA HIV-1 DNA | 11 | 695428 | 705028 | 1 | 4.91E-05 | - | Yes | Yes | 3.2717e-310 | Yes | rs7113204; rs2613996; rs12366210; rs7113255 |
| ENSG00000177156 | *TALDO1* | RNA:DNA ratio, CA HIV-1 DNA | 11 | 747329 | 765024 | 1 | 0.000176 | - | Yes | Yes | 2.53E-06 | Yes | rs7113204; rs2613996; rs12366210; rs7113255 |
| ENSG00000177225 | *PDDC1* | RNA:DNA ratio, CA HIV-1 DNA | 11 | 767220 | 777488 | -1 | 0.61729 | -1.49614 | Yes | No | - | Yes | rs7113204; rs2613996; rs12366210; rs7113255 |
| ENSG00000255284 | *AP006621.5* | RNA:DNA ratio, CA HIV-1 DNA | 11 | 777578 | 784297 | 1 | - | - | Yes | Yes | 8.69E-19 | Yes | rs7113204; rs2613996; rs12366210; rs7113255 |
| ENSG00000184524 | *CEND1* | RNA:DNA ratio, CA HIV-1 DNA | 11 | 787104 | 790123 | -1 | 0.584353 | -0.06781 | Yes | Yes | 1.88E-05 | Yes | rs7113204; rs2613996; rs12366210; rs7113255 |
| ENSG00000177542 | *SLC25A22* | RNA:DNA ratio, CA HIV-1 DNA | 11 | 790475 | 798316 | -1 | 0.568406 | -0.32739 | Yes | Yes | 1.35E-06 | Yes | rs7113204; rs2613996; rs12366210; rs7113255 |
| ENSG00000177595 | *PIDD* | RNA:DNA ratio, CA HIV-1 DNA | 11 | 799179 | 809753 | -1 | 1.6E-09 | - | Yes | Yes | 4.81E-10 | Yes | rs7113204; rs2613996; rs12366210; rs7113255 |
| ENSG00000177600 | *RPLP2* | RNA:DNA ratio, CA HIV-1 DNA | 11 | 809647 | 812880 | 1 | 0.708518 | -0.75387 | Yes | No | - | Yes | rs7113204; rs2613996; rs12366210; rs7113255 |
| ENSG00000177666 | *PNPLA2* | RNA:DNA ratio, CA HIV-1 DNA | 11 | 818902 | 825573 | 1 | 0.001141 | -0.58581 | Yes | Yes | 5.49E-08 | Yes | rs7113204; rs2613996; rs12366210; rs7113255 |
| ENSG00000177685 | *EFCAB4A* | RNA:DNA ratio, CA HIV-1 DNA | 11 | 826144 | 831991 | 1 | 0.001859 | 0.62083 | Yes | Yes | 3.02E-12 | Yes | rs7113204; rs2613996; rs12366210; rs7113255 |
| ENSG00000177697 | *CD151* | RNA:DNA ratio, CA HIV-1 DNA | 11 | 832843 | 839831 | 1 | 0.001643 | - | Yes | Yes | 7.88E-17 | Yes | rs7113204; rs2613996; rs12366210; rs7113255 |
| ENSG00000177700 | *POLR2L* | RNA:DNA ratio, CA HIV-1 DNA | 11 | 837356 | 842545 | -1 | 0.532881 | - | Yes | Yes | 1.18E-05 | No | rs7113204; rs2613996; rs12366210; rs7113255 |
| ENSG00000214063 | *TSPAN4* | RNA:DNA ratio, CA HIV-1 DNA | 11 | 842808 | 867116 | 1 | 2.52E-06 | -0.35345 | Yes | No | - | No | rs7113204; rs2613996; rs12366210; rs7113255 |
| ENSG00000177830 | *CHID1* | RNA:DNA ratio, CA HIV-1 DNA | 11 | 867357 | 915058 | -1 | 0.000726 | -1.18508 | Yes | Yes | 0.000166 | Yes | rs7113204; rs2613996; rs12366210; rs7113255 |
| ENSG00000183020 | *AP2A2* | RNA:DNA ratio, CA HIV-1 DNA | 11 | 924894 | 1012239 | 1 | 0.999932 | 0.093705 | Yes | No | - | No | rs7113204; rs2613996; rs12366210; rs7113255 |
| ENSG00000184956 | *MUC6* | RNA:DNA ratio, CA HIV-1 DNA | 11 | 1012821 | 1036706 | -1 | 1.09E-32 | -0.43461 | Yes | No | - | No | rs7113204; rs2613996; rs12366210; rs7113255 |
| ENSG00000198788 | *MUC2* | RNA:DNA ratio | 11 | 1074875 | 1104419 | 1 | 4.55E-05 | - | Yes | No | - | No | rs12366210 |
| ENSG00000215182 | *MUC5AC* | RNA:DNA ratio | 11 | 1151580 | 1222364 | 1 | - | - | Yes | No | - | No | rs12366210 |

## Table S3. QTL SNPs affect the expression of identified loci

| **HIV Measurement** | **Variant** | **Estimated Allele** | **Effect on HIV Measurement** | **Gene** | **P value (eQTL)** | **Effect on Gene Expression** |
| --- | --- | --- | --- | --- | --- | --- |
| CA HIV-1 DNA | rs2613996 | A | + | PTDSS2 | 3.1E-50 | + |
| RNA:DNA ratio | rs7113204 | G | - | RNH1 | 9.83E-10 | - |
|  |  |  |  | PTDSS2 | 1.48E-15 | + |
|  |  |  |  | IRF7 | 0.000000336 | + |
|  | rs12366210 | G | - | DEAF1 | 7.52E-13 | + |
|  | rs7817589 | C | + | RP11-1149M10.2 | 3.84E-14 | - |

## Table S4. Publicly available AIDS GWAS QTLs estimated in current study

| **HIV measurement** | **SNP rsID** | **Chr.** | **Base-pair Position** | **HIV measurement beta** | **HIV measurement P-value** | **GWAS study link** | **GWAS study title** | **GWAS study SNP gene** | **GWAS study P-value** | **GWAS study odds ratio or beta** | **GWAS traits** |
| --- | --- | --- | --- | --- | --- | --- | --- | --- | --- | --- | --- |
| CA HIV-1 DNA | rs1015164 | 3 | 46410189 | -0.094912145 | 0.030518845 | www.ncbi.nlm.nih.gov/pubmed/21502085 | Genome-wide association study implicates PARD3B-based AIDS restriction. | ENSG00000121797 | 0.000009 | 1.46 | AIDS |
| CA HIV-1 DNA | rs1015164 | 3 | 46410189 | -0.094912145 | 0.030518845 | www.ncbi.nlm.nih.gov/pubmed/26553974 | Polymorphisms of large effect explain the majority of the host genetic contribution to variation of HIV-1 virus load. | ENSG00000121797 | 2E-19 |  | HIV-1 infection, HIV viral set point measurement |
| CA HIV-1 DNA | rs1015164 | 3 | 46410189 | -0.094912145 | 0.030518845 | www.ncbi.nlm.nih.gov/pubmed/31219150 | Single nucleotide polymorphisms in HLA alleles are associated with HIV-1 viral load in demographically diverse, ART-naïve participants from the START trial. | ENSG00000121797 | 1E-15 | 0.23 | HIV-1 infection, viral load |
| CA HIV-1 DNA | rs2883821 | 1 | 18240357 | -0.206229721 | 0.006096871 | www.ncbi.nlm.nih.gov/pubmed/26148204 | Genomewide association study of tenofovir pharmacokinetics and creatinine clearance in AIDS Clinical Trials Group protocol A5202. | ENSG00000117154 | 0.000003 | - | Response to tenofovir, HIV infection, creatinine clearance measurement |
| CA HIV-1 DNA | rs2883821 | 1 | 18240357 | -0.206229721 | 0.006096871 | www.ncbi.nlm.nih.gov/pubmed/31219150 | Single nucleotide polymorphisms in HLA alleles are associated with HIV-1 viral load in demographically diverse, ART-naïve participants from the START trial. | ENSG00000117154 | 1E-15 | 0.23 | HIV-1 infection, viral load |
| CA HIV-1 DNA | rs7691759 | 4 | 9686390 | 0.110854106 | 0.041639289 | www.ncbi.nlm.nih.gov/pubmed/31219150 | Single nucleotide polymorphisms in HLA alleles are associated with HIV-1 viral load in demographically diverse, ART-naïve participants from the START trial. | ENSG00000287117 | 3E-16 | 0.21 | HIV-1 infection, viral load |
| CA HIV-1 DNA | rs3131018 | 6 | 31175805 | 0.123760279 | 0.003716242 | www.ncbi.nlm.nih.gov/pubmed/21051598 | The major genetic determinants of HIV-1 control affect HLA class I peptide presentation. | ENSG00000223364, ENSG00000224056, ENSG00000226422, ENSG00000230983, ENSG00000224744, ENSG00000231450, ENSG00000204528, ENSG00000204531, ENSG00000233911, ENSG00000229094, ENSG00000230336, ENSG00000206454, ENSG00000237582, ENSG00000235068 | 4E-16 | 2.1 | HIV-1 infection |
| CA HIV-1 DNA | rs9264942 | 6 | 31306603 | 0.092243564 | 0.022130757 | www.ncbi.nlm.nih.gov/pubmed/20041166 | Common genetic variation and the control of HIV-1 in humans. | ENSG00000228964, ENSG00000223532, ENSG00000234745, ENSG00000232126, ENSG00000224608, ENSG00000206450 | 6E-32 | 5.3 | HIV-1 infection |
| CA HIV-1 DNA | rs9264942 | 6 | 31306603 | 0.092243564 | 0.022130757 | www.ncbi.nlm.nih.gov/pubmed/20041166 | Common genetic variation and the control of HIV-1 in humans. | ENSG00000228964, ENSG00000223532, ENSG00000234745, ENSG00000232126, ENSG00000224608, ENSG00000206450 | 6E-12 | - | HIV-1 infection |
| CA HIV-1 DNA | rs9264942 | 6 | 31306603 | 0.092243564 | 0.022130757 | www.ncbi.nlm.nih.gov/pubmed/21051598 | The major genetic determinants of HIV-1 control affect HLA class I peptide presentation. | ENSG00000228964, ENSG00000223532, ENSG00000234745, ENSG00000232126, ENSG00000224608, ENSG00000206450 | 3E-35 | 2.9 | HIV-1 infection |
| RNA:DNA ratio | rs7568498 | 2 | 161172602 | -0.115440864 | 0.023535339 | www.ncbi.nlm.nih.gov/pubmed/24554482 | Genome-wide association study of peripheral neuropathy with D-drug-containing regimens in AIDS Clinical Trials Group protocol 384. | ENSG00000136560 | 0.00000002 | - | Response to reverse transcriptase inhibitor, HIV-1 infection, peripheral neuropathy |
