# Supplementary figures and images for: IRF7 and RNH1 are modifying factors of HIV-1 reservoirs: a genome-wide association analysis
Source: BMC Med. 2021 Nov 16;19:282. doi: 10.1186/s12916-021-02156-5 (PMC8594146; doi:10.1186/s12916-021-02156-5)

Figure S1

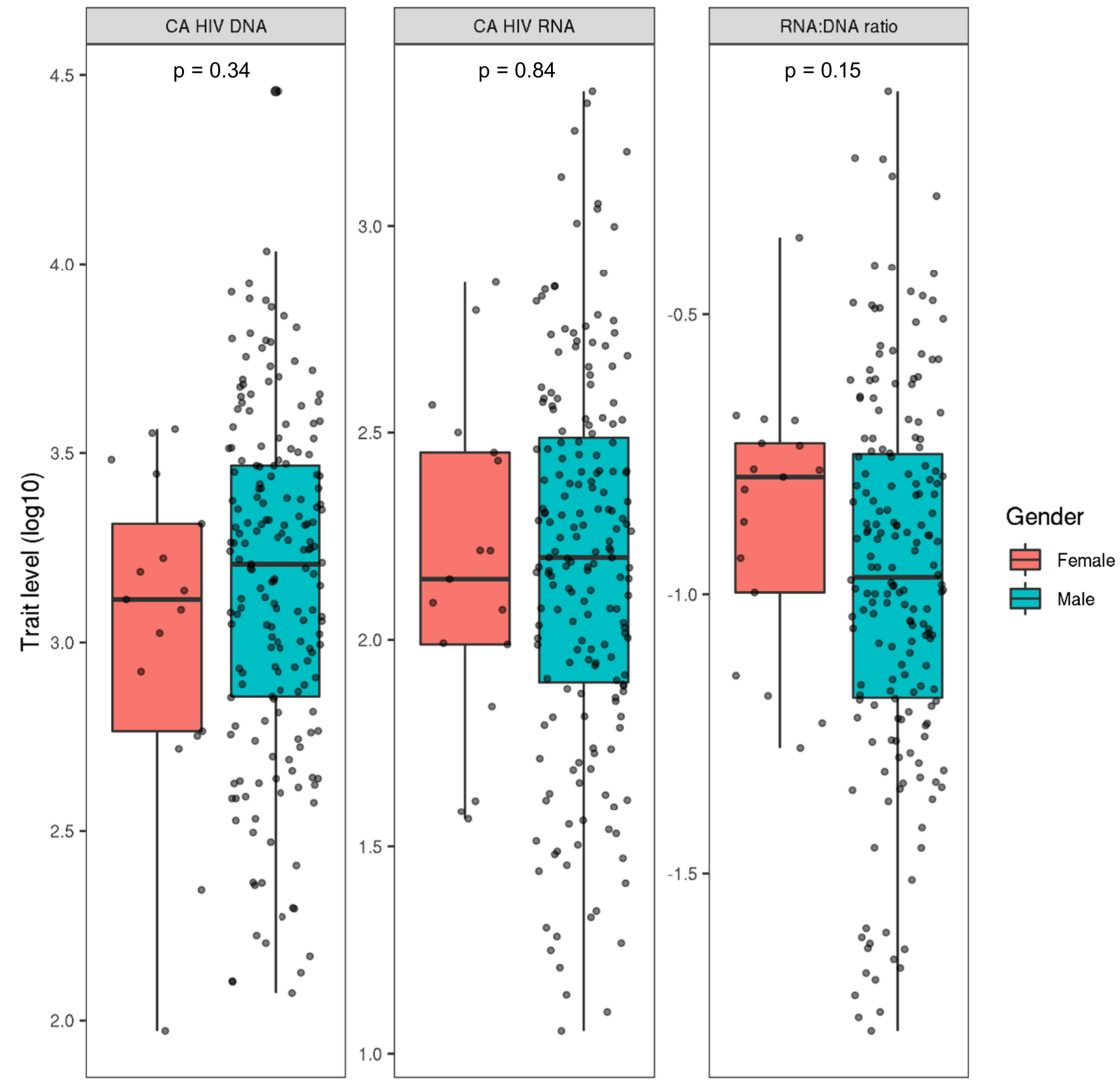

Figure S2

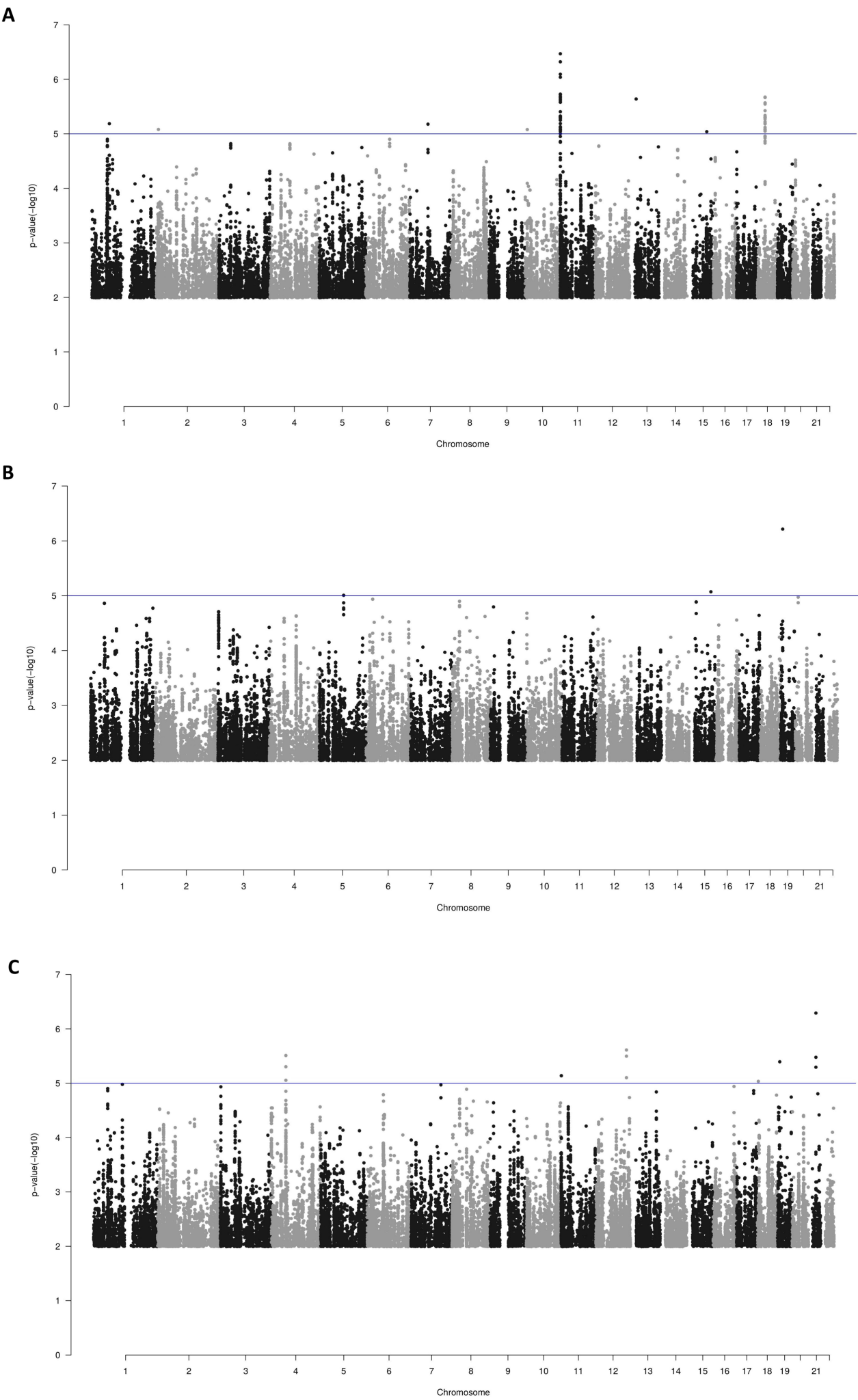

Figure S3

A

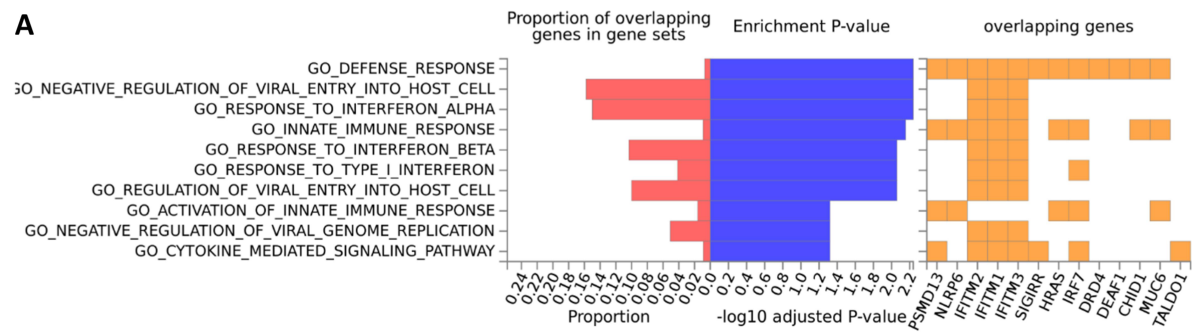

B

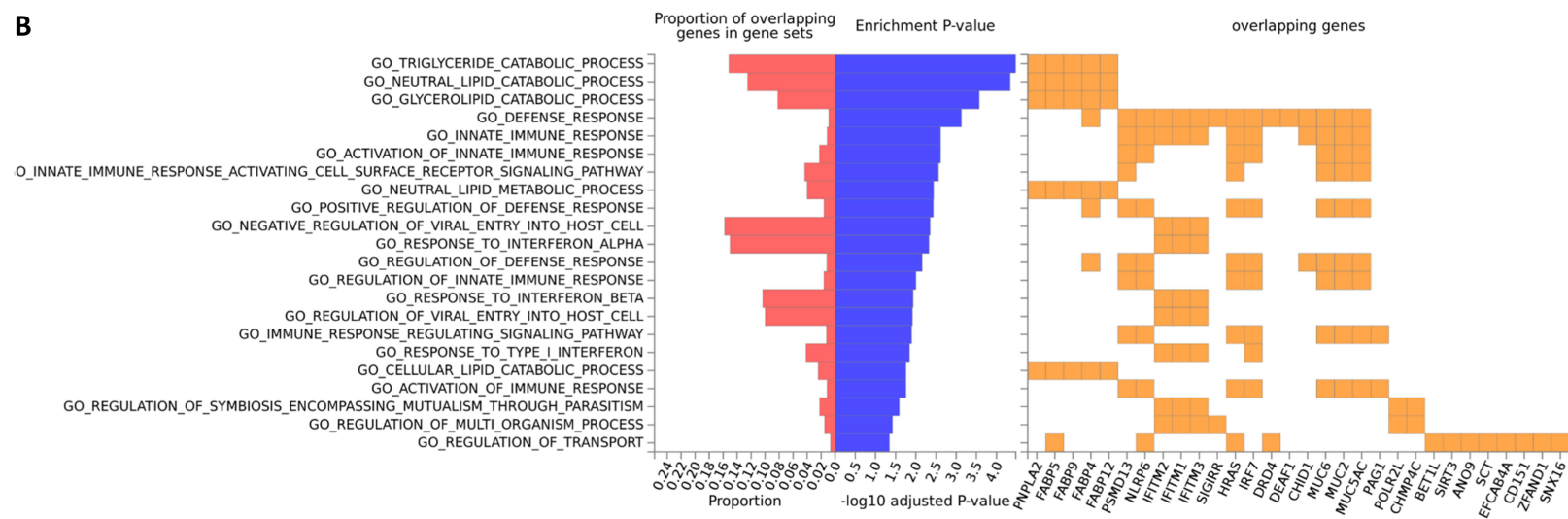

Figure S4

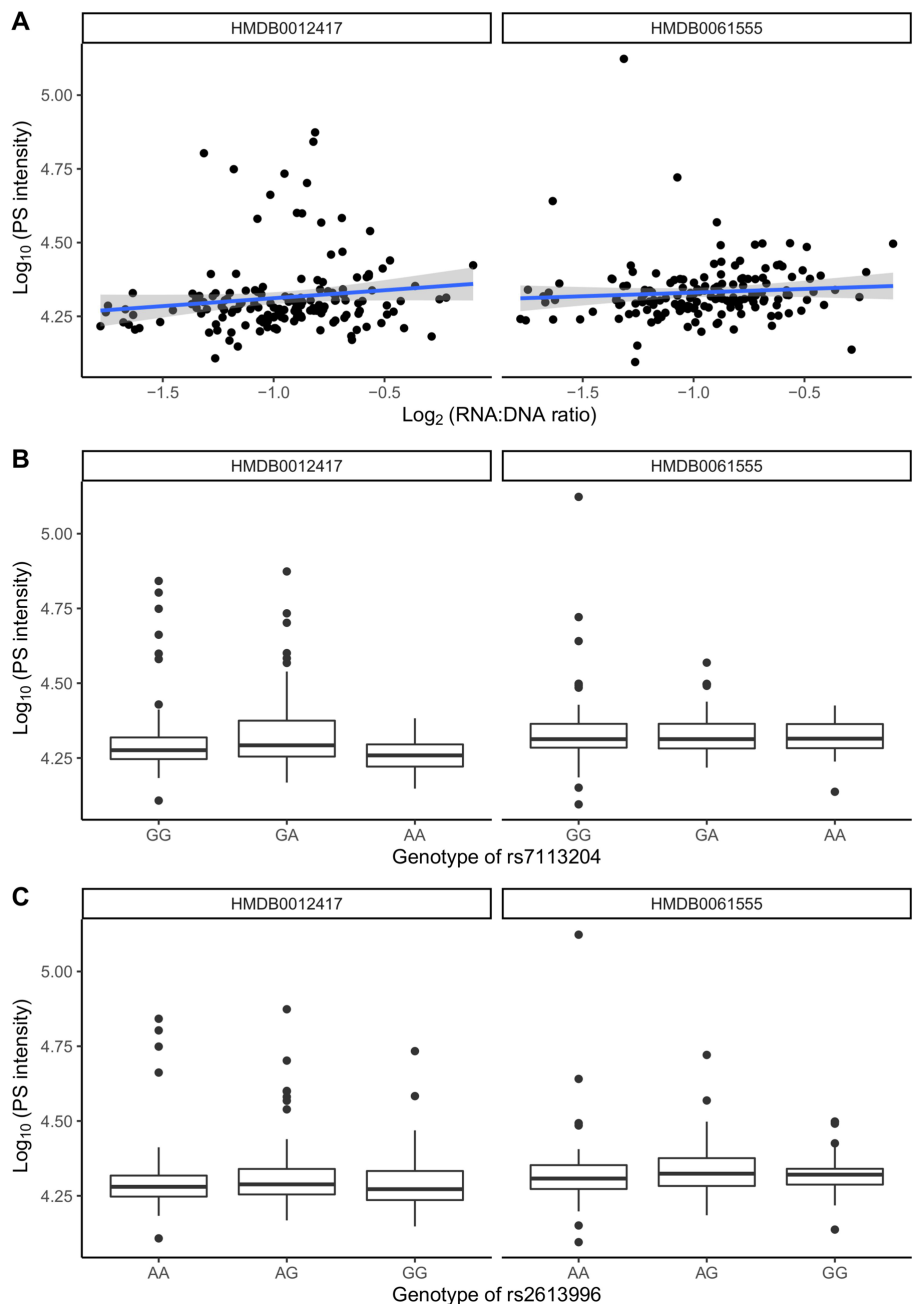

Figure S5

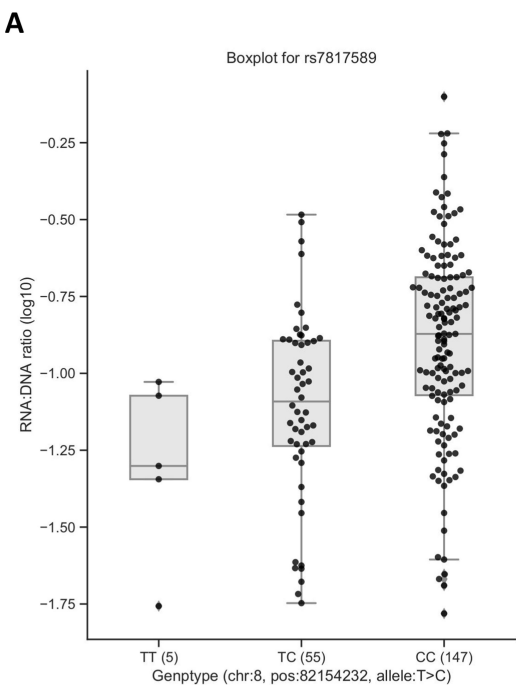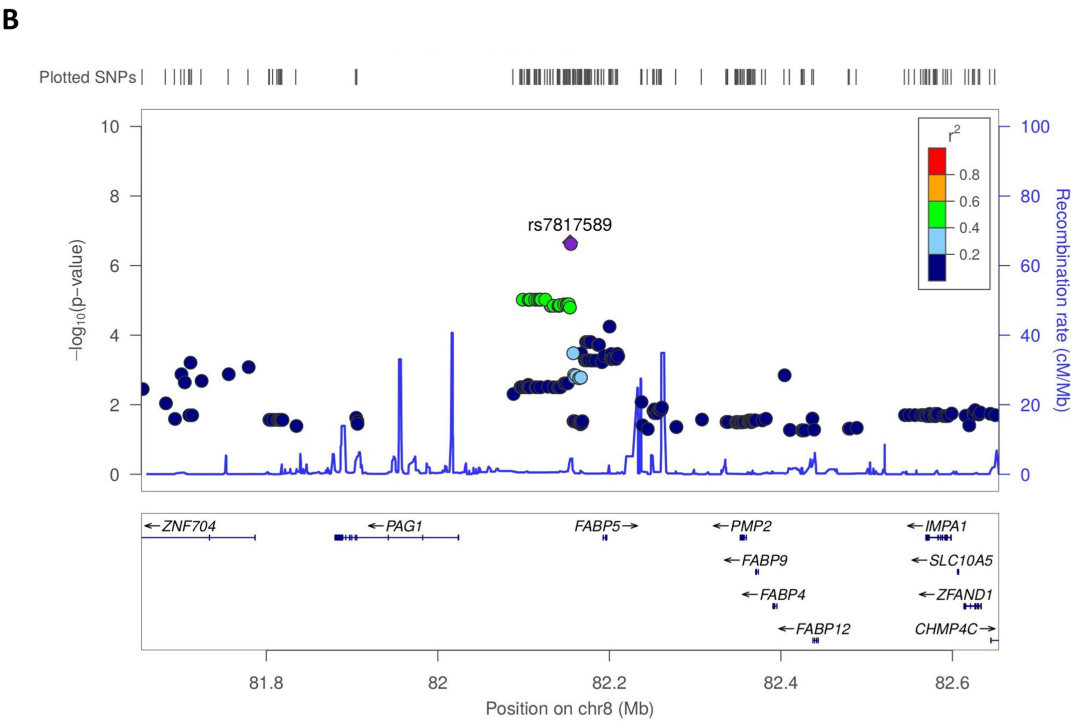

Figure S6

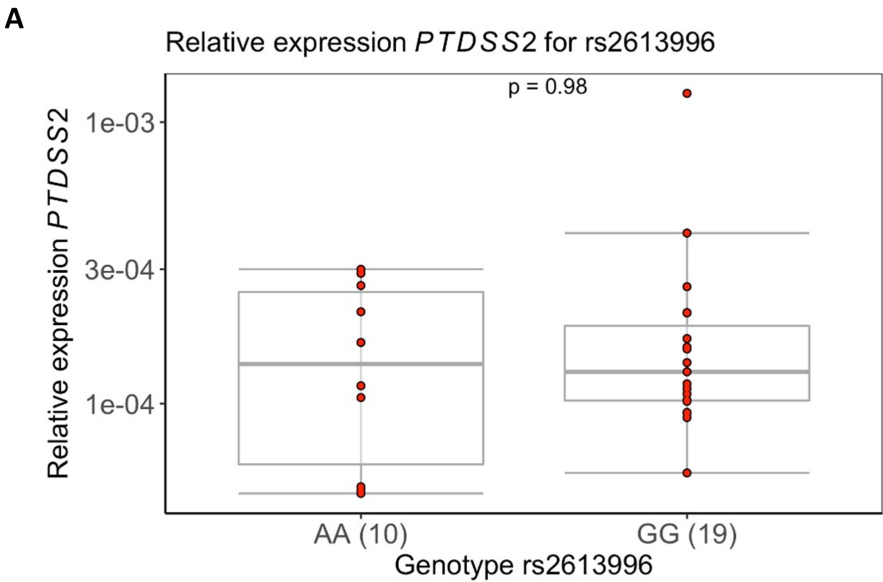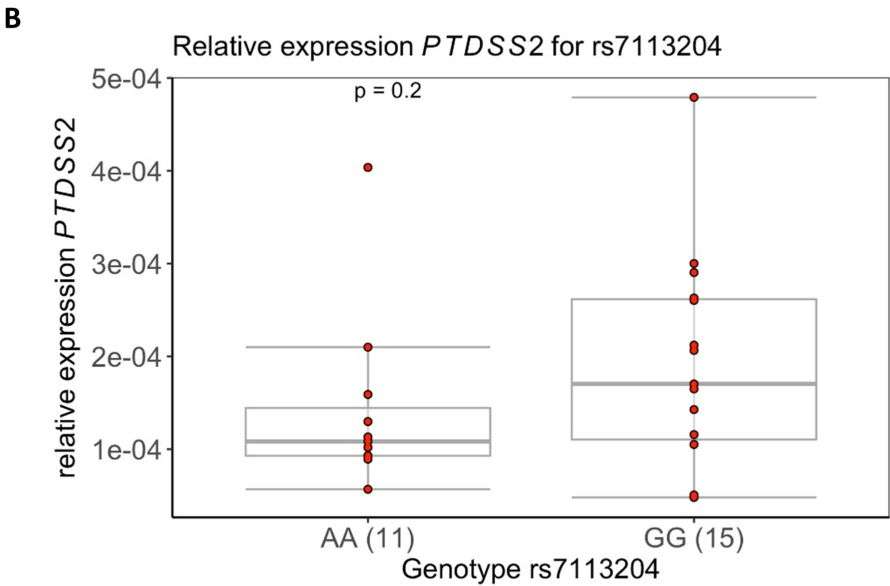

Figure S7

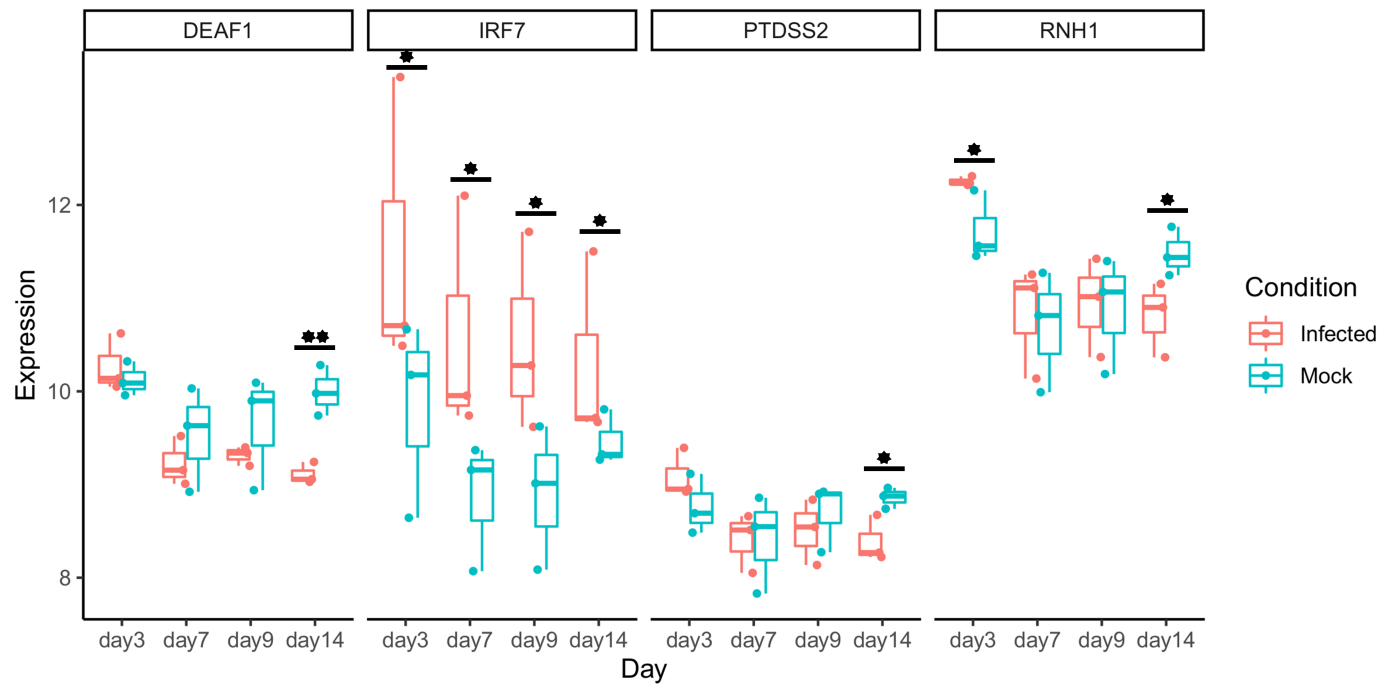

Figure S8

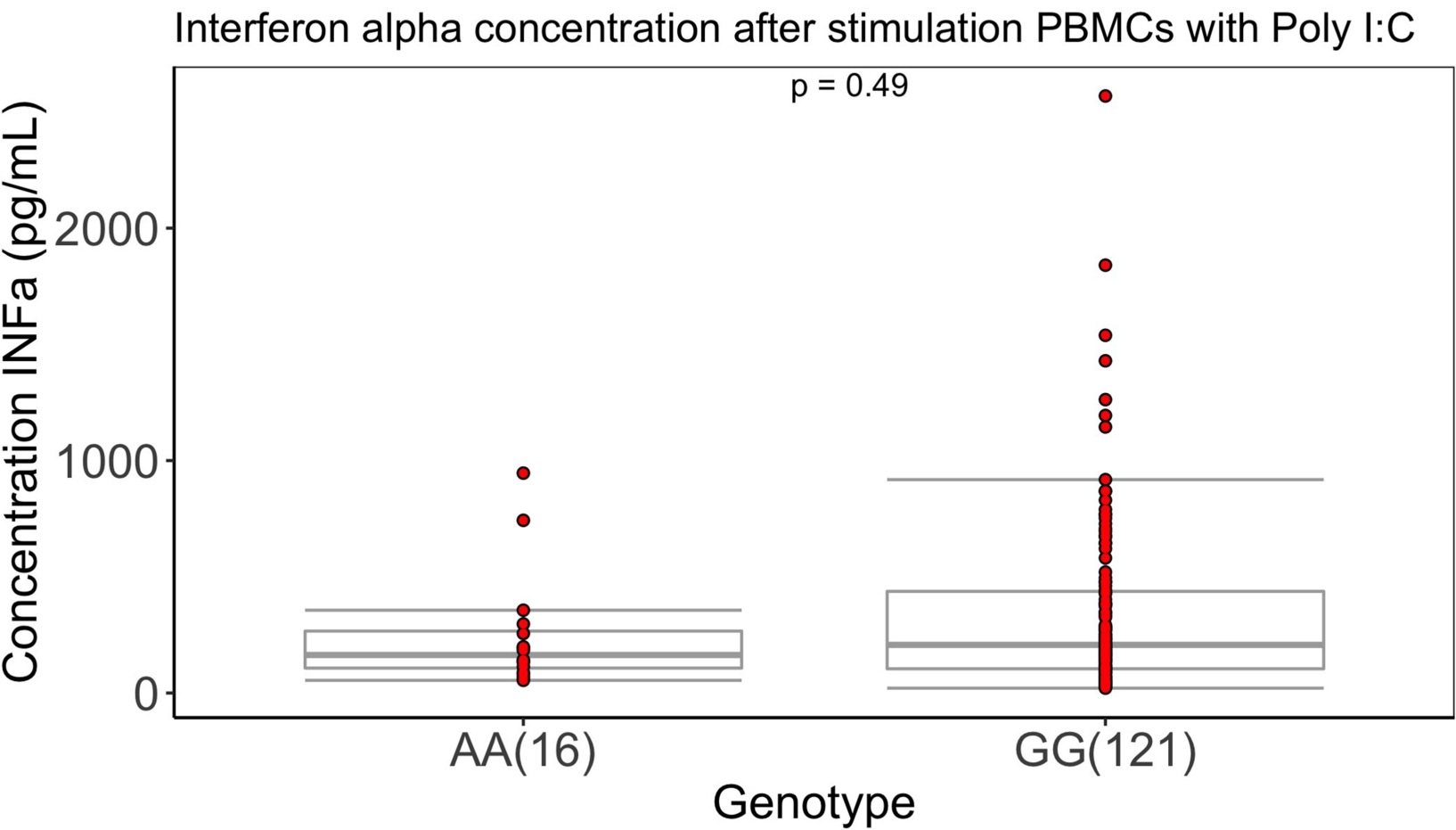

Supplement: Supplementary file 2 — Additional file 2: Figure S1. Three panels of the figure are respective differences of CA HIV-1 RNA, CA HIV-1 DNA, and RNA:DNA ratio between male and female. However, by a student test, sex does not affect any of the three traits at the threshold of P < 0.05. Figure S2. A, B, and C are Manhattan plots for CA HIV-1 DNA, CA HIV-1 RNA, and CA HIV-1 RNA (adjusted by CA HIV-1 DNA). However, no association was found at the threshold of P < 5 × 10^-7^. Moreover, all the associative analyses have an inflation factor close to 1. Figure S3. (A) Gene Ontology (biological process) annotation and enrichment of genes at locus identified as genome-wide significantly associated with CA HIV-1 DNA. (B) Gene Ontology (biological process) annotation and enrichment of genes at locus identified as genome-wide suggestively associated with RNA:DNA ratio. Figure S4. (A) Correlation between two types of PS and RNA:DNA ratio. The horizontal axis represents the Log2 transformed RNA:DA ratio. The vertical axis represents the PS intensity transformed by Log10. (B) PS intensity transformed by Log10 per genotype of rs7113204. No significant difference among three genotypes was found for each PS. (C) PS intensity transformed by Log10 per genotype of rs2613996. No significant difference among three genotypes was found for each PS. Figure S5. (A) Box plot for each genotype of rs7817589 from QTL mapping analysis for RNA:DNA ratio. This locus is identified as suggestively associated with the trait at the threshold of P = 2.17 × 10^-7^. (B) LocusZoom plot of the top SNP of QTL mapping analysis for RNA:DNA ratio on chromosome 8. Figure S6. (A) Box plot of relative expression of PTDSS2 compared in a subset of subjects with variant rs2613996-A (n = 10) compared to subjects with variant rs2613996-G (n = 19). (B) Box plot of relative expression of PTDSS2 in subjects with SNP rs7113204-A (n = 11) compared to SNP rs7113204-G (n = 14). Data in these box plots are visualized in log transformation. Groups are compare [file 12916_2021_2156_MOESM2_ESM.pdf]
